# Perceptions and practices among students, parents, and teachers of the Human Papillomavirus (HPV) Vaccination: A pilot feasibility study from Rural Kerala

**DOI:** 10.1371/journal.pone.0340559

**Published:** 2026-08-10

**Authors:** Anjum John, Roshna Rasheed, Mercy John Idikula

**Affiliations:** 1 Department of Community Medicine, Pushpagiri Institute of Medical Sciences & Research Center, Tiruvalla, Kerala, India; 2 Department of Microbiology, Pushpagiri Institute of Medical Sciences & Research Center, Tiruvalla, Kerala, India; Federal University Otuoke, NIGERIA

## Abstract

**Background:**

India bears the world’s highest cervical cancer burden, yet awareness and uptake of the effective HPV vaccine remain low. Although Kerala has initiated pilot HPV vaccination programmes, stakeholder knowledge, attitudes, and practices (KAP) are not well documented. This pilot study aimed to assess the feasibility of a KAP study and to identify gaps in the questionnaire, and refine study procedures (tool and process refinement) for a future large-scale analytical study.

**Methods:**

A cross-sectional pilot study was conducted in a school in Pulinkunnu, Kerala, among students aged 11–15 years, their parents, and teachers. A structured, validated questionnaire, developed in English, translated into Malayalam, was administered during school hours. Responses were entered into secure electronic spreadsheets. Descriptive statistics were used to summarise questionnaire responses and assess the feasibility, clarity, and performance of study items. As this pilot study was designed primarily to refine study procedures and measurement tools rather than test hypotheses, no inferential or multivariable analyses were performed.

**Results:**

The pilot included 75 students and 20 teachers; no parents participated despite planned recruitment, highlighting an important feasibility challenge for the proposed full-scale study. Among participants, females constituted the majority. HPV-related knowledge was low across groups, with widespread misconceptions and limited awareness of vaccine availability and cervical cancer screening. Attitudes toward vaccination were mixed, marked by safety concerns and modest willingness to receive or recommend the vaccine. Practices reflected low prior awareness and uptake, with few respondents having heard of or received the HPV vaccine.

**Conclusions:**

This pilot revealed major gaps in HPV-related knowledge, attitudes, and practices among participating students and teachers, while also identifying important methodological challenges, particularly parental recruitment and questionnaire refinement. The findings support modifications to recruitment strategies, study procedures, and measurement tools before implementation of a larger analytical study.

## Introduction

Human Papillomavirus (HPV) is the most common sexually transmitted infection globally, with around 40 strains associated with infections of the anogenital region. Although most of these infections are self-limiting, certain high-risk strains are linked to cancers such as cervical, anal, oropharyngeal, and penile cancer. [1] Despite the availability of effective vaccines, HPV-related diseases remain a major public health concern, particularly in low- and middle-income countries where screening and vaccination coverage are limited. Understanding public knowledge, attitudes, and practices (KAP) regarding HPV infection and HPV vaccination is therefore essential for informing evidence-based prevention strategies and improving vaccine uptake. [2–5]

### Public health burden of HPV

Globally, cervical cancer is the fourth most common cancer among women. In 2023, it was estimated that there were 604,000 new cervical cancer cases, causing around 342,000 deaths worldwide. Projections suggest an annual increase of nearly 100,000 new cases between 2018 and 2030. Prevalence of HPV is influenced by social and economic determinants like gender inequality and poverty, with illiteracy and early sexual initiation contributing to over 85% of cervical cancer cases, with an additional risk in people living with Human Immunodeficiency virus (HIV) infections. [6,7] The disease threatens the well-being of children when mothers die prematurely due to cervical cancer. [8]

### Disparities in healthcare

Cervical cancer is a preventable and curable disease. Despite the availability of the HPV vaccine for the prevention of cervical cancer, and effective treatment measures- much of the world has limited access to these services. Deaths due to cervical cancer are three times higher in low- or lower- middle- income countries, demonstrating the widespread disparities in the availability, accessibility, and adaptability of health care services in regions with the highest disease burden. [9]

Integrating HPV vaccination, cervical cancer screening, and management of precancerous lesions/cervical cancer into health systems remain essential. Involving people in their own healthcare helps reduce the incidence, mortality, and burden of HPV-related conditions. [10] The demonstrated success of HPV vaccination has prompted a global commitment to eliminate cervical cancer as a public health problem. [11]

### Global strategy

Public health microbiology is at the threshold of a rare but doable feat- the elimination of a non-communicable disease- cervical cancer. The World Health Organization’s 2020 Global Strategy to eliminate cervical cancer set the 90-70-90 targets by 2030: vaccinate 90% of girls under 15 years, screen 70% of women at 35 and 45 years of age, and treat 90% of women identified with cervical disease. [7,11]

### HPV in India

India has the highest global burden of cervical cancer. Annually, about 132,000 new cases and 80,000 deaths occur, with a high prevalence of HPV type 16. [5,6] The vastness of the country and the diversity of its population are responsible for the lack of proper documentation of the epidemiology and patterns of HPV virus strains. [10] Thus, as in other lower income countries, the implementation of prevention and control programs has been difficult. This highlights the importance of HPV vaccination in India.

### Vaccine coverage in India and Kerala

India licensed two HPV vaccines in 2008. The state of Sikkim successfully implemented the first school-based immunization program in 2018, achieving >95% coverage with strong government and community support. [12] In 2022, the National Technical Advisory Committee on Immunization recommended introducing the indigenously developed quadrivalent vaccine into the Universal Immunization Program (UIP). The vaccine rollout was planned for 2024, with Kerala’s Wayanad and Alappuzha districts included in the pilot phase. [13–16] In Kerala, voluntary uptake remains poorly documented, and some studies report declining cervical cancer rates (7–9/100,000 women annually), which has sometimes been used to argue for screening rather than vaccination. [17] A 2018 study found only 8% coverage among medical students. [18] More recently, the Happy Noolpuzha Initiative in Wayanad demonstrated local-level success by vaccinating tribal girls through a Panchayat-led campaign. [19] In recent months, the HPV vaccination was included into India’s national immunization strategy.

### Review of literature

Prior studies in India show low levels of HPV awareness and vaccination. Backer et al. (2024) found that although 85.6% of college students in Kerala knew HPV causes cervical cancer, only 48.6% had heard of the vaccine, and uptake was low (11.6% in medical students, 4.9% in non-medical). [20] Basu et al. similarly reported limited uptake among adolescents in North India, citing lack of awareness, cost, and cultural hesitancy. [21] School-based vaccination and awareness campaigns have proven effective in increasing acceptance. [22–24] However, teachers and parents often lack adequate knowledge, with safety concerns and stigma acting as major barriers. [25–27] Although several studies from India have examined HPV awareness among college students and healthcare trainees, evidence regarding school-going adolescents, their parents, and teachers remains limited, particularly in Kerala where there is an ongoing statewide implementation of HPV vaccination programmes.

### Rationale for the present study

Limited evidence exists on the knowledge, attitudes, and practices of school-going adolescents, parents, and teachers—key school-based stakeholders in vaccine acceptance. This gap limits the development of effective, school-based prevention strategies, despite ongoing HPV vaccination initiatives. Additionally, no validated tools were available to assess HPV-related knowledge and perceptions in this setting, necessitating the development and pilot testing of a new questionnaire. This pilot study was therefore undertaken to evaluate the feasibility of the tool, study item clarity and completeness of variables, and ensure that the instrument could adequately capture stakeholder perspectives before implementing a full-scale study. By identifying gaps in awareness, acceptance, and questionnaire performance, this pilot aimed to generate preliminary insights to guide a larger, more comprehensive investigation. The protocol for this study was published in the Indian Journal of Applied Research in October 2025.(https://doi.org/10.36106/ijar and ISSN No: 2249-555X available from (https://www.worldwidejournals.com/indian-journal-of-applied-research-(IJAR)/fileview/knowledge-attitude-and-practice-regarding-human-papillomavirus-hpv-vaccine-among-school-students-parents-and-teachers-in-central-kerala-a-crosssectional-study-protocol_October_2025_5111739822_7103697.pdf)

## Objectives

The study was conducted to assess the feasibility of measuring knowledge, attitudes, and practices related to HPV and HPV vaccination among school students aged 11–15 years, their teachers, and their parents, in preparation for a larger study.

## Materials and methods

### Study design and setting

A pilot cross-sectional study was conducted in 2025 at a higher secondary school in Pulinkunnu, Alappuzha, Kerala, targeting students aged 11–15 years, their teachers, and their parents. This study was explicitly designed as a pilot to refine instruments, assess feasibility, and inform changes to the questionnaire and resource scale up, consistent with recommended guidelines for pilot and feasibility studies. [28,29]

### Study participants

All students aged 11–15 years enrolled in the selected school and present on the day of data collection were eligible to participate, subject to informed assent and parental consent. Teachers of the selected classes and parents of enrolled students were also invited to participate.

### Sample size and sampling

As this was a pilot study, universal sampling was employed. All students aged 11–15 years enrolled in the selected school, their teachers, and parents constituted the sampling frame.

## Tools

A brief structured questionnaire was developed with close-ended questions to assess the perceptions and practices related to HPV infection and vaccination. (Refer S1 File – Original questionnaire) Given the exploratory objectives of the pilot, sociodemographic information was not collected. A single, common questionnaire was used for both parents and teachers to ensure uniformity and reduce respondent burden. Questionnaire items were informed by a review of existing KAP studies on HPV vaccination and were framed using simple, non-judgmental language to enhance comprehension and participation. Content validity was assessed through review by subject experts from the departments of Community Medicine and Biostatistics, who evaluated each item for relevance, clarity, and appropriateness for the target population. The questionnaire was developed in English and translated into Malayalam using a forward–backward translation process. Forward translation was performed by a bilingual professional fluent in both English and Malayalam. The translated version was independently back-translated into English by another bilingual expert blinded to the original questionnaire. Discrepancies between the original and back-translated versions were reviewed by experts to ensure semantic, conceptual, and cultural equivalence. The pre-final Malayalam version was pre-pilot tested testing among a small group of postgraduate students and subject experts, to assess time taken to completion, clarity, acceptability, and ease of answering questions. Feedback from this process led to minor refinements, primarily aimed at shortening the questionnaire and improving wording. Based on feedback and expert review, necessary modifications were made before finalizing the questionnaire for the main pilot study.

Internal consistency and reliability of the questionnaire was assessed during pilot instrument testing using Cronbach’s alpha. The overall questionnaire demonstrated acceptable internal consistency (Cronbach’s α = 0.73). Domain-specific alpha coefficients were 0.63 for the knowledge domain, 0.65 for the attitude domain, and 0.24 for the practice domain. These estimates were derived from a small preliminary pilot sample and were used primarily to identify items requiring refinement prior to implementation of the main pilot feasibility study.

### Data collection

Trained investigators administered the survey to students and teachers during school hours using paper-based forms. Parents were provided questionnaires through their wards. All completed forms were anonymized, coded, and entered into secure, password-protected electronic spreadsheets.

### Recruitment period

Data was collected between the 18^th^ and 19^th^ of June, 2025. Parental consent was taken on the first day, with the questionnaires and information sheet about the research sent home with the students for parental review. Student assent was taken on the second day before the questionnaires were filled in by them. Teacher consent was taken during the day of the study.

### Outcome measures

The pilot study assessed the feasibility, practicality, and clarity of items measuring knowledge of HPV and cervical cancer, awareness of the HPV vaccine, attitudes toward vaccination, willingness to receive or recommend the vaccine, perceived barriers, vaccination-related practices, and sources of information. These outcomes were used primarily to evaluate the performance of the questionnaire and identify areas requiring refinement for the follow up full-scale study.

### Data analysis

Completed questionnaires were reviewed for completeness at the time of collection, Data were entered into password-protected electronic spreadsheets and cross-checked against the original forms to minimise data-entry errors before analysis.

Data were cleaned and analysed using descriptive statistics to summarize sociodemographic characteristics and KAP outcomes. With no key demographic or behavioural variables available for multivariable modelling, no inferential or regression analyses were undertaken. The focus of analysis was to evaluate response patterns, identify missing or non-discriminating items, and assess the overall feasibility and suitability of the questionnaire for use in a larger study.

### Ethics statement

The study protocol was approved by the Institutional Review Board of Pushpagiri Institute of Medical Sciences and Research Centre (PIMSRC/E1/388A/101/2024). Information sheets and consent forms were provided to parents through participating students prior to data collection. Parents were invited to participate in the survey; however, no completed parent questionnaires were returned. This recruitment challenge was documented as an important feasibility finding of the pilot study and will inform enhanced parent-engagement strategies in the larger study.

Written informed consent was obtained from participating teachers. For students, parental permission for student participation was obtained through consent forms distributed before data collection, and verbal assent was obtained from each student immediately before questionnaire administration. Verbal student assent was obtained from all participating students after providing age-appropriate information about the study, and documented by study investigators in participant enrolment records. Participation was voluntary, and all responses were anonymised before analysis.

## Results

### Demographic profile and recruitment feasibility

The study was conducted at an Indian Certificate of School Education (ICSE) school at Pulinkunnu, Alleppey district, Kerala, India and included 75 students and 20 teachers. Although parents were identified as a target stakeholder group, no completed parent questionnaires were returned despite distribution through students, representing a deviation from the planned study design. Table 1 shows the age classification of the study participants.

**Table 1:** Age classification of the students and teachers.

Age Group	Frequency	Percentage
10-12 years	34	45.3%
13-15 years	27	36%
16-17 years	14	18.7%
	**75**	
Teachers		
30-39 years	6	30%
40-49 years	8	40%
50-59 years	6	30%
	**20**	

Among the students, 16 (21.3%) were boys and 59 (78.7%) were girls. Among teachers, 2 (10%) were male and 18 (90%) were female. (Table 1)

### Knowledge regarding HPV vaccination

Knowledge regarding HPV infection, cervical cancer, and HPV vaccination is presented in Table 2.

**Table 2:** Knowledge regarding HPV infection, cervical cancer prevention, and HPV vaccination among students and teachers. Values are presented as frequencies, percentages, and 95% confidence intervals. Students (n = 75); Teachers (n = 20). (Refer S1 Table. Item-level comparison of HPV-related knowledge among students and teachers.).

Question	Answer options	Students	Teachers
K1.HPV infection can cause cervical cancer	Yes	33 (44.0%) [32.5–55.9]	10 (50.0%) [27.2–72.8]
	No	42 (56.0%) [44.1–67.5]	10 (50.0%) [27.2–72.8]
	Don’t know	0	0
K2.HPV is a sexually transmitted infection.	Yes	38 (50.7%) [38.9–62.4]	9 (45.0%) [23.1–68.5]
	No	37 (49.3%) [37.6–61.1]	11 (55.0%) [31.5–76.9]
	Don’t know	0	0
K3. HPV affects only women.	Yes	38 (50.7%) [38.9–62.4]	6 (30.0%) [11.9–54.3]
	No	37 (49.3%) [37.6–61.1]	14 (70.0%) [45.7–88.1]
	Don’t know	0	0
K4. The HPV vaccine is most effective when given before sexual debut.	Yes	35 (46.7%) [35.1–58.6]	12 (60.0%) [36.1–80.9]
	No	40 (53.3%) [41.4–64.9]	8 (40.0%) [19.1–63.9]
	Don’t know	0	0
K5. The HPV vaccine is available in India.	Yes	35 (46.7%) [35.1–58.6]	13 (65.0%) [40.8–84.6]
	No	40 (53.3%) [41.4–64.9]	7 (35.0%) [15.4–59.2]
	Don’t know	0	0
K6. The HPV vaccine protects against all types of HPV.	Yes	40 (53.3%) [41.4–64.9]	7 (35.0%) [15.4–59.2]
	No	35 (46.7%) [35.1–58.6]	13 (65.0%) [40.8–84.6]
	Don’t know	0	0
K7. Boys can also receive the HPV vaccine	Yes	39 (52.0%) [40.2–63.6]	9 (45.0%) [23.1–68.5]
	No	36 (48.0%) [36.4–59.8]	11 (55.0%) [31.5–76.9]
	Don’t know	0	0
K8. The HPV vaccine requires more than one dose.	Yes	36 (48.0%) [36.4–59.8]	15 (75.0%) [50.9–91.3]
	No	39 (52.0%) [40.2–63.6]	5 (25.0%) [8.7–49.1]
	Don’t know	0	0
K9. HPV infection can occur even without symptoms	Yes	40 (53.3%) [41.4–64.9]	9 (45.0%) [23.1–68.5]
	No	35 (46.7%) [35.1–58.6]	11 (55.0%) [31.5–76.9]
	Don’t know	0	0
K10. Pap smear is a screening test for cervical cancer.	Yes	37 (49.3%) [37.6–61.1]	8 (40.0%) [19.1–63.9]
	No	38 (50.7%) [38.9–62.4]	12 (60.0%) [36.1–80.9]
	Don’t know	0	0
K11. Do you know where to get the HPV vaccine?	Yes	40 (53.3%) [41.4–64.9]	0 (0.0%) [0.0–16.8]
	No	35 (46.7%) [35.1–58.6]	20 (100.0%) [83.2–100.0]

Knowledge regarding vaccine availability, dosing schedules, screening methods, and vaccine access varied among both groups.

### Attitudes toward HPV vaccination

Attitudes toward HPV vaccination among students and teachers are presented in Table 3. Refer to S2 Table for Item-level comparison of attitudes towards HPV vaccination among students and teachers for further details.

**Table 3:** Attitudes towards HPV vaccination among students and teachers. Values are presented as frequencies, percentages, and 95% confidence intervals. Students (n = 75); Teachers (n = 20).

Attitude Questions	Answer Options	Students	Teachers
A1. HPV vaccination should be included in the national immunization schedule.	Strongly Agree	15(20%) [11.8–31]	2 (10.0%) [1.2–31.7]
	Agree	15(20%) [11.8–31]	6 (30.0%) [11.9–54.3]
	Neutral	19 (25.3%) [16.0–37.1]	4 (20.0%) [5.7–43.7]
	Disagree	15 (20.0%) [11.8–31.2]	4 (20.0%) [5.7–43.7]
	Strongly Disagree	11 (14.7%) [7.6–24.7]	4 (20.0%) [5.7–43.7]
A2. I believe HPV vaccination is important for cancer prevention.	Strongly Agree	11 (14.7%) [7.6–24.7]	2 (10.0%) [1.2–31.7]
	Agree	12(16.0%) [8.5-26.7]	4 (20.0%) [5.7–43.7]
	Neutral	16 (21.3%) [12.8–32.2]	3 (15.0%) [3.2–37.9]
	Disagree	15 (20.0%) [11.8–31.2]	5 (25.0%) [8.7–49.1]
	Strongly Disagree	21 (28.0%) [18.3–39.6]	6 (30.0%) [11.9–54.3]
A3. I would recommend the HPV vaccine to others.	Strongly Agree	15 (20.0%) [11.8–31.2]	8 (40.0%) [19.1–63.9]
	Agree	18 (24.0%) [14.9–35.3]	4 (20.0%) [5.7–43.7]
	Neutral	10 (13.3%) [6.6–23.2]	3 (15.0%) [3.2–37.9]
	Disagree	17 (22.7%) [13.8–34.5]	2 (10.0%) [1.2–31.7]
	Strongly Disagree	14 (18.7%) [10.6–29.3]	3 (15.0%) [3.2–37.9]
A4. I worry about the side effects of the HPV vaccine.	Strongly Agree	19 (25.3%) [16.0–37.1]	14 (18.7%) [10.6–29.3]
	Agree	14 (18.7%) [10.6–29.3]	8 (40.0%) [19.1–63.9]
	Neutral	17 (22.7%) [13.8–34.5]	3 (15.0%) [3.2–37.9]
	Disagree	10 (13.3%) [6.6–23.2]	2 (10.0%) [1.2–31.7]
	Strongly Disagree	15 (20.0%) [11.8–31.2]	4 (20.0%) [5.7–43.7]
A5. HPV vaccination promotes early sexual activity	Strongly Agree	13 (17.3%) [9.5–28.0]	4 (20.0%) [5.7–43.7]
	Agree	11 (14.7%) [7.6–24.7]	2 (10.0%) [1.2–31.7]
	Neutral	20 (26.7%) [17.2–38.1]	5 (25.0%) [8.7–49.1]
	Disagree	11(14.7%) [7.6-24.7]	2 (10.0%) [1.2–31.7]
	Strongly Disagree	20 (26.7%) [17.2–38.1]	7 (35.0%) [15.4–59.2]
A6. I believe awareness campaigns are needed for HPV vaccination.	Strongly Agree	14 (18.7%) [10.6–29.3]	1 (5.0%) [0.1–24.9]
	Agree	13 (17.3%) [9.5–28.0]	3 (15.0%) [3.2–37.9]
	Neutral	17(22.7%) [13.8-34.5]	8 (40.0%) [19.1–63.9]
	Disagree	13 (17.3%) [9.5–28.0]	5 (25.0%) [8.7–49.1]
	Strongly Disagree	18 (24.0%) [14.9–35.3]	3 (15.0%) [3.2–37.9]
A7. I trust the safety of the HPV vaccine.	Strongly Agree	3 (15.0%) [3.2–37.9]	1 (5.0%) [0.1–24.9]
	Agree	13 (17.3%) [9.5–28.0]	3 (15.0%) [3.2–37.9]
	Neutral	17 (22.7%) [13.8-34.5]	8 (40.0%) [19.1–63.9]
	Disagree	13 (17.3%) [9.5–28.0]	5 (25.0%) [8.7–49.1]
	Strongly Disagree	18 (24.0%) [14.9–35.3]	3 (15.0%) [3.2–37.9]
A8. If not vaccinated, are you willing to take it?	Yes	33 (44.0%) [32.5–55.9]	8 (40.0%) [19.1–63.9]
	No	42 (56.0%) [44.1–67.5]	12 (60.0%) [36.1–80.9]

Responses were distributed across all attitude categories. Neutral responses were common for several items. Beliefs regarding HPV vaccination and early sexual activity, trust in vaccine safety, and the need for awareness campaigns showed varied responses across both groups.

### Practices related to HPV vaccination

Practice-related responses are presented in Table 4. Refer to S3 Table for Item-level comparison of HPV vaccination practices among students and teachers for more information.

**Table 4:** HPV vaccine awareness, uptake, and recommendation practices among students and teachers. Values are presented as frequencies, percentages, and 95% confidence intervals. Students (n = 75); Teachers (n = 20).

Practice Questions	Answer Options	Students	Teachers
P1. Have you heard about the HPV vaccine before?	Yes	45 (60.0%) [48.9–71.1]	10 (50.0%) [27.2–72.8]
	No	30 (40.0%) [28.9–51.1]	10 (50.0%) [27.2–72.8]
P2. Have you ever received the HPV vaccine?	Yes	39 (52.0%) [40.2–63.6]	10 (50.0%) [27.2–72.8]
	No	36(48.0%) [36.4-59.8]	10 (50.0%) [27.2–72.8]
P3. Have you encouraged anyone to take the HPV vaccine?	Yes	30 (40.0%) [28.9–51.1]	11 (55.0%) [31.5–76.9]
	No	45 (60.0%) [48.9–71.1]	9 (45.0%) [23.1–68.5]

### Comparison of Domain Scores Between Students and Teachers

Comparison of overall domain scores between students and teachers showed no statistically significant differences. The mean knowledge score was 5.48 (95% CI: 5.06–5.90) among students and 4.90 (95% CI: 4.16–5.64) among teachers (p = 0.169).The mean attitude score was 22.32 (95% CI: 21.38–23.26) among students and 21.35 (95% CI: 19.91–22.79) among teachers (p = 0.253).The mean practice score was 2.49 (95% CI: 2.27–2.72) among students and 2.35 (95% CI: 1.89–2.81) among teachers (p = 0.568).

## Discussion

This pilot feasibility school-based study was primarily designed to evaluate recruitment procedures, questionnaire performance, and the practicality of assessing HPV-related knowledge, attitudes, and practices among students, parents, and teachers in a rural Kerala school setting. When examined alongside the national and global evidence base, the results highlight systemic information and knowledge gaps, attitudinal barriers, sociocultural influences, widespread misconceptions, and structural and methodological barriers that limit HPV vaccine acceptability and uptake in India. The substantial chasms in awareness, and low vaccination uptake despite moderate exposure to HPV-related information can inform the design of future school-based HPV vaccination research and interventions. However, the findings should be interpreted cautiously and viewed as preliminary observations rather than population-level estimates. A summary of the study findings is presented in Supporting Information S4 Table.

### Knowledge of HPV infection and cervical cancer prevention

The study suggests that the understanding of HPV transmission and cervical cancer prevention modalities remains incomplete among both students and teachers. Only about half of students and teachers recognised that HPV was a sexually transmitted infection that causes cervical cancer. These findings align with several other Indian studies demonstrating poor foundational knowledge about HPV. Backer et al. (2024) reported that although 85.6% of Kerala college students knew HPV was linked to cervical cancer, only 48.6% had heard of the HPV vaccine. [20] Similar patterns were seen in North India, where Basu et al. found that adolescents were largely unaware of HPV transmission and its association with cervical cancer. [21] Internationally, limited awareness is well-documented in Low Middle-Income Countries (LMIC), particularly where sexual health education is restricted, limited, or inconsistently delivered. [30,31]

Knowledge regarding cervical cancer screening was limited in the study population, particularly among the teachers studied. This indicates that many participants may not fully appreciate that both vaccination and screening encompass the continuum of cervical cancer prevention. Such knowledge gaps may reflect the limited integration of HPV-related health education within school curricula and emphasises the importance of strengthening teacher preparedness for school-based HPV vaccination initiatives.

Overall, the knowledge gap in our study is consistent with national trends and highlights the absence of integrated HPV education in schools, even in high-literacy states like Kerala.

### Awareness of HPV Vaccination and Vaccine Access

Awareness of HPV vaccination and vaccine availability was limited among study participants. Many respondents were unaware that the HPV vaccine was available in India or from where it could be obtained. Similar findings have been reported in other studies involving healthcare trainees and university students, where uncertainty regarding vaccine availability and access remained common despite awareness of cervical cancer prevention. [32]

In contrast, regions that have implemented structured school-based HPV vaccination programmes have reported substantially higher levels of vaccine awareness. For example, Sikkim’s successful HPV vaccination programme was preceded by intensive stakeholder engagement and health education activities that improved awareness among students, parents, teachers, and the wider community. [12] The contrast suggests that although Kerala has achieved notable progress in health and education indicators, systematic communication regarding HPV vaccination has not yet been implemented at a comparable scale. The findings therefore highlight the need for targeted awareness campaigns addressing vaccine availability, access pathways, and eligibility for vaccination among all stakeholders.

### Attitudes towards HPV vaccination

The predominance of “neutral” or “uncertain” attitudes in this study indicates “ambivalent” hesitancy or ‘uncertainty” rather than outright opposition or refusal. Neutral responses across several attitude questions may demonstrate limited confidence in applying existing knowledge. These may have figured as predominant barriers to HPV vaccine acceptance in this setting. Similar findings are widely observed in global vaccine acceptance research. Lelliott et al. describe “ambivalence” as a form of “information hesitancy,” where individuals are unsure due to incomplete knowledge rather than opposition. [33] Backer et al. and Muthukrishnan et al.’s studies confirms that unclear, inconsistent, or conflicting information contributes to passive resistance to vaccination. [20,34]

Safety concerns were common in our sample, consistent with a recent study by Waheed et al., and an earlier study by Holman, which identified perceived risk of adverse events as the most influential barrier to HPV vaccination globally. [35,36] Similar concerns have influenced attitudes towards HPV vaccine acceptance in studies from India and other LMIC settings. Misinformation and limited discussion of sexual health among the populace create sociocultural norms surrounding adolescent sexual health, contributing to vaccine hesitancy.

Conversely, countries with long-standing HPV vaccination programmes—such as Australia and the United Kingdom—report significantly lower safety-related hesitancy because of the strong public trust and robust adverse event monitoring. [37,38] Importantly, studies have shown that the willingness to be vaccinated was relatively high once individuals were informed. This finding is encouraging because it suggests that hesitancy may be modifiable through education rather than reflecting entrenched opposition to vaccination, Asmelash et al. demonstrated that even brief educational interventions significantly increase willingness to vaccinate. [39] Similar improvements have been documented in US and European settings. [40] Targeted interventions that provide accurate information regarding vaccine safety, effectiveness, and long-term cancer prevention benefits may therefore improve vaccine confidence and facilitate greater acceptance among students, teachers, and families.

Thus, the results of our study suggest that structured school-based awareness programmes could improve vaccine acceptance in the setting of Pulinkunnu.

### Practices and the Knowledge–Behaviour Gap

Despite moderate awareness, actual vaccination uptake remained low among both students and teachers. Although a proportion of respondents had heard of the HPV vaccine and expressed willingness to receive it, relatively few reported previous vaccinations or encouraged others to be vaccinated. The discrepancy observed highlights the complexity of vaccine decision-making, and is well documented in India.

Basu et al. found that even when adolescents expressed interest, parental concerns, cost, and lack of access remained barriers. [21] Das et al. reported low uptake even among medical students, demonstrating that knowledge in some settings does not directly translate to action. [18]

The absence of parental participation in our study further highlights a major barrier. Numerous studies have shown that parental consent and beliefs are the strongest predictors of adolescent HPV vaccination. In South India, for example, parental attitudes accounted for more than 50% of vaccination decisions. [27,41] The complete absence of parental responses here underscores the urgent need to involve families in HPV awareness initiatives.

When compared with successful models, the reasons for the gap becomes clearer. Sikkim’s programme and the Happy Noolpuzha campaign in Wayanad achieved high coverage through community mobilisation, strong local leadership, and parent-centred communication. [12,19] Our findings suggest that without community engagement, school-based programmes alone may be insufficient.

The vaccination practice gaps may reflect multiple barriers, including concerns about vaccine safety, limited awareness regarding vaccine availability, inadequate communication from healthcare providers and schools, and broader sociocultural discomfort surrounding discussions of sexually transmitted infections. Such findings suggest that improving knowledge alone may be insufficient and that interventions addressing accessibility, trust, and communication may also be required.

### Misconceptions and underlying influences

A major misconception observed was the belief that HPV affects only women. This misconception has been highlighted globally. Studies from Europe and the United States show that boys often underestimate their susceptibility to HPV-related diseases, including penile, oropharyngeal, and anal cancers. [42,43] This gendered perception reduces motivation for male vaccination, despite WHO and CDC recommendations for universal vaccination of both sexes. [44–46]

Fear of HPV vaccine side effects was prominent in our study, echoing findings from several Indian and global studies. Seo et al. reported through their scoping review that concerns about safety and adverse events are the leading reason for HPV vaccine refusal among parents. [47] Asmelash et al. similarly identified safety concerns as a major determinant of vaccine hesitancy across West and East African countries. [39] Such concerns are often fuelled by misinformation, lack of transparent communication, and limited exposure to scientific evidence.

Another recurring misconception was the belief that HPV vaccination may promote early sexual activity. This has been repeatedly shown to be untrue in scientific literature, yet remains a widespread cultural fear. Studies in Türkiye, Africa, and South Korea report that parents worry vaccination may be interpreted as permission for sexual activity. [48–50] Studies from the US, however, show no association between HPV vaccination and sexual behaviour. [51] The persistence of this misconception highlights cultural discomfort with sexuality education, particularly in conservative settings.

Misconceptions about the number of doses required and uncertainty about asymptomatic HPV infections further indicate the absence of structured, accurate health communication in schools. Similar misunderstandings have been reported in East Asian and African countries, where lack of clarity about vaccine schedules, HPV transmission, and the role of screening hinders adherence, reflecting limited integration of HPV-related health education within school settings, before they can support or advocate for school-based vaccination initiatives. [49,52–54]

### Influence of participant characteristics and teacher preparedness

The study population was predominantly female among both students and teachers. This may have influenced perceptions of HPV and HPV vaccination, particularly because cervical cancer prevention has traditionally been framed as a women’s health issue. Although Kerala has a female-favouring population structure, the proportion of female participants in this study exceeded that expected from the general population and may limit the generalisability of the findings.

Most participating teachers were in mid-career age groups and represented an important stakeholder group for future school-based HPV vaccination initiatives. Despite their potential role as sources of health information and advocates for adolescent vaccination, several knowledge gaps and misconceptions were identified among teachers. These findings suggest that teacher-focused educational interventions and capacity-building programmes may be necessary before implementing large-scale school-based HPV vaccination campaigns.

The most important feasibility finding of this pilot study was the complete absence of parental participation. As parents are key decision-makers regarding adolescent vaccination, their non-participation limits the comprehensiveness of the findings but simultaneously provides valuable information regarding the challenges of engaging this stakeholder group.

### Lessons learned and proposed changes for future research

The pilot demonstrated that while the questionnaire was able to describe levels of knowledge, attitudes, and self-reported practices regarding HPV vaccination, it was unable to identify meaningful determinants of these outcomes. Important contextual variables operating at the parental, household, school, and socioeconomic levels—including parental educational status, household decision-making processes, prior exposure to HPV-related information, healthcare access, and other sociodemographic characteristics—were either absent or inadequately captured. Consequently, although the study provided useful descriptive insights, it highlighted the need to incorporate these variables into the planned revision of the questionnaire to enable exploration of contextual variables, factors associated with HPV vaccine-related knowledge, attitudes, and practices, and complementary qualitative methods in the planned larger study.

a. Add socioeconomic and demographic variables that can act as predictors

Sociodemographic predictors like parental level of education, occupation and socioeconomic status, family structure and decision-maker for child vaccination in families, and prior exposure to health campaigns or school health programs are important predictors which must be studied. Specific health system and information exposure questions need to be explored such as the source of HPV vaccine information, level of trust in healthcare providers and government programs, previous experience with childhood or adolescent vaccinations, or awareness of government HPV vaccination policies. Cultural and belief-based factors are another dimension for study, like perceived stigma around sexually transmitted infections, comfort discussing sexual health topics, perceived moral or cultural concerns related to HPV vaccination, gender norms influencing adolescent health decisions.

b Modify data-collection mode

The pilot indicated that self-administered online questionnaires may not be optimal, particularly for parents, as literacy and digital familiarity may affect response quality. The following methodological changes are being proposed: interviewer-administered, short, semi-structured questionnaires for parents and teachers where possible to explore reasoning behind practices and capture unanticipated concerns or motivations. These would enhance data completeness, depth, and validity.

c. Failure of Parent Recruitment

The most significant feasibility challenge identified during the pilot was the complete absence of parental participation despite distribution of questionnaires through students. As parents are the primary decision-makers regarding adolescent vaccination, their perspectives are essential for understanding HPV vaccine acceptance and uptake. The pilot demonstrated that relying solely on student-mediated questionnaire distribution is insufficient for engaging parents. Future studies should employ more direct recruitment strategies, including parent–teacher meetings, reminder notices, telephone follow-up, electronic surveys, community engagement activities, and interviewer-administered questionnaires to improve parental participation.

d. Need to Explore Household Decision-Making Processes

The pilot questionnaire did not adequately capture how vaccination decisions are made within households. Decisions regarding adolescent vaccination are often influenced by multiple family members, including parents, grandparents, and other caregivers. Future studies should include questions examining who makes health-related decisions, the degree of parental involvement in vaccination choices, sources of advice consulted before vaccination, and the influence of family beliefs and cultural norms. Understanding these processes will be essential for designing effective communication strategies.

e. Need for Assessment of Teacher Preparedness and Training

Teachers represent a critical stakeholder group in school-based vaccination programmes and often serve as trusted sources of information for students and parents. The pilot revealed important knowledge gaps among teachers regarding HPV infection, cervical cancer prevention, and HPV vaccination. Future studies should therefore include dedicated questions assessing previous training, confidence in discussing HPV-related topics, perceived educational needs, and willingness to participate in school-based health promotion activities. Such information will help identify training requirements before implementation of HPV vaccination programmes.

f. Need to Assess Exposure to Health Information

The pilot identified limited awareness regarding HPV vaccination and vaccine availability but did not investigate where participants obtained health information from. Future questionnaires should assess exposure to information from schools, healthcare providers, social media, traditional media, family members, peers, and community organisations. Understanding the channels through which individuals receive information will help identify opportunities for targeted educational interventions and public health messaging.

g. Need for Mixed-Methods Approaches

The findings demonstrated that quantitative questionnaires alone may not fully explain the reasons underlying vaccine hesitancy, misconceptions, and decision-making. Issues such as concerns about vaccine safety, perceptions of adolescent sexuality, trust in healthcare systems, and cultural influences are often complex and context-specific. Future studies should incorporate qualitative methods such as focus group discussions and in-depth interviews with students, parents, teachers, and healthcare providers. These approaches would provide richer insights into the beliefs, experiences, and contextual factors that shape HPV vaccine acceptance.

h. Need for Improved Assessment of Vaccine Access and Uptake Barriers

Although the pilot identified limited awareness and uptake of HPV vaccination, the questionnaire did not comprehensively assess barriers to vaccine access. Future versions should include detailed questions regarding vaccine availability, affordability, distance to vaccination centres, healthcare provider recommendations, parental concerns, logistical challenges, and perceived barriers within schools and communities. Such information will help distinguish knowledge-related barriers from structural barriers to vaccination and support the development of targeted interventions.

Overall, these lessons highlight the value of the pilot study in identifying weaknesses in recruitment procedures, questionnaire design, and contextual data collection. Addressing these issues before conducting a larger study will enhance both the scientific validity and practical relevance of future research on HPV vaccination in Kerala.

### Public Health Implications

The findings of this study highlight the requirement for three strategic actions. School based health education with structured sessions for teachers, parents, and students addressing safety, immunisation schedules, benefits, costs, adverse events, and availability of the HPV are required. Parental involvement with regular meetings, and open house sessions with educational brochures for clarifying doubts are recommended. The places where HPV vaccines are available, the methods to procure the vaccine etc should be provided to ensure accessibility and clarity for referral. These strategies align with WHO’s elimination framework and the national plan for HPV vaccination under the Universal Immunisation Program (UIP).

### Strengths of the pilot study

A key strength of this pilot study lies in its feasibility-driven design. Conducted as a preliminary investigation, the study was essential for refining the student, teacher, and parent questionnaires, particularly to identify and restructure items capturing key predictor variables for HPV vaccine knowledge, attitudes, and acceptance. The use of a validated questionnaire, combined with insights gained from pilot testing, enhanced content clarity, contextual relevance, and construct coverage for the main study.

The transparent reporting of methodological challenges and feasibility issues encountered during implementation is a strength of the study. By documenting recruitment failures, questionnaire limitations, and missing contextual variables, the study provides valuable lessons for future researchers and contributes to improving the design and conduct of school-based HPV vaccination research.

The pilot study conducted among 75 students and 20 teachers in a socio-demographically comparable setting—the neighbouring district of Alappuzha (district neighbouring to Pathanamthitta district where the full study is planned)—allowed assessment of recruitment processes, participation rates, and data-collection logistics without contaminating the main study population. It also provided realistic estimates of time, personnel, and material resources required for large-scale implementation, thereby strengthening the methodological preparedness of the primary study.

### Limitations

Limitations include the absence of parent responses during the pilot phase, the single-school setting, and reliance on self-reported practices, which may be subject to reporting bias. Online or self-filled formats (e.g., Google Forms) were associated with incomplete responses, superficial answers, and difficulty probing reasoning behind decisions. Although Kerala has a female-favouring sex ratio of 1,084 females per 1,000 males, the study sample contained a substantially higher proportion of female students and teachers. This imbalance may have influenced responses regarding HPV-related knowledge, attitudes, and practices and limits the generalizability of the findings.

However, consistent with the purpose of pilot and feasibility studies, the findings are not intended to be generalisable but to inform study design and implementation. The study hitherto provides valuable baseline insights from a rural context relevant to HPV vaccine introduction.

## Conclusion

The study demonstrates major knowledge gaps, mixed attitudes, and low HPV vaccination uptake among students and teachers, though willingness improves when informed. Strengthening school-based awareness, involving parents, and improving access to vaccination services will be critical for successful programme implementation. With national HPV rollout underway, these findings can guide local action for cervical cancer prevention.

### Patient and public involvement and disclaimer

Students, teachers, and parents were not involved in the development of the research protocol or the pilot questionnaire. As this was a preliminary feasibility study, no direct involvement in interpreting or disseminating the results is planned at this stage. Insights from the pilot will instead be used internally by the research team to refine the study tools and procedures for the full-scale investigation.

## Supporting information

S1 Table Item-level comparison of HPV-related knowledge among students and teachers. (DOCX)

S2 Table Item-level comparison of attitudes towards HPV vaccination among students and teachers. (DOCX)

S3 Table Item-level comparison of HPV vaccination practices among students and teachers. (DOCX)

S4 Table Summary of key findings and implications from the pilot feasibility study on HPV vaccination knowledge, attitudes, and practices. (DOCX)

S1 File Original questionnaire. (DOCX)

S2 File Modified questionnaire. (DOCX)

S3 File De-identified dataset. (XLSX)
