# Supplementary material for: Perceptions and practices among students, parents, and teachers of the Human Papillomavirus (HPV) Vaccination: A pilot feasibility study from Rural Kerala
Source: PLoS One. 2026 Aug 10;21(8):e0340559. doi: 10.1371/journal.pone.0340559 (PMC13456282; doi:10.1371/journal.pone.0340559)
Supplement: Supplementary file 1 [file pone.0340559.s001.docx]

S1 Table. Item-level comparison of HPV-related knowledge among students and teachers

| Question | Answer options | Students( % (95% CI) | Teachers % (95% CI) | p-value |
| --- | --- | --- | --- | --- |
| K1.HPV infection can cause cervical cancer | Yes | 33 (44.0%) [32.5–55.9] | 10 (50.0%) [27.2–72.8] | 0.632 |
|  | No | 42 (56.0%) [44.1–67.5] | 10 (50.0%) [27.2–72.8] |  |
|  | Don’t know | 0 | 0 |  |
| K2.HPV is a sexually transmitted infection. | Yes | 38 (50.7%) [38.9–62.4] | 9 (45.0%) [23.1–68.5] | 0.652 |
|  | No | 37 (49.3%) [37.6–61.1] | 11 (55.0%) [31.5–76.9] |  |
|  | Don’t know | 0 | 0 |  |
| K3. HPV affects only women. | Yes | 38 (50.7%) [38.9–62.4] | 6 (30.0%) [11.9–54.3] | 0.100 |
|  | No | 37 (49.3%) [37.6–61.1] | 14 (70.0%) [45.7–88.1] |  |
|  | Don’t know | 0 | 0 |  |
| K4. The HPV vaccine is most effective when given before sexual debut. | Yes | 35 (46.7%) [35.1–58.6] | 12 (60.0%) [36.1–80.9] | 0.289 |
|  | No | 40 (53.3%) [41.4–64.9] | 8 (40.0%) [19.1–63.9] |  |
|  | Don’t know | 0 | 0 |  |
| K5. The HPV vaccine is available in India. | Yes | 35 (46.7%) [35.1–58.6] | 13 (65.0%) [40.8–84.6] | 0.145 |
|  | No | 40 (53.3%) [41.4–64.9] | 7 (35.0%) [15.4–59.2] |  |
|  | Don’t know | 0 | 0 |  |
| K6. The HPV vaccine protects against all types of HPV. | Yes | 40(53.3%)[41.4-64.9] | 7 (35.0%) [15.4–59.2] | 0.145 |
|  | No | 35 (46.7%) [35.1–58.6] | 13 (65.0%) [40.8–84.6] |  |
|  | Don’t know | 0 | 0 |  |
| K7. Boys can also receive the HPV vaccine | Yes | 39 (52.0%) [40.2–63.6] | 9 (45.0%) [23.1–68.5] | 0.578 |
|  | No | 36 (48.0%) [36.4–59.8] | 11 (55.0%) [31.5–76.9] |  |
|  | Don’t know | 0 | 0 |  |
| K8. The HPV vaccine requires more than one dose. | Yes | 36 (48.0%) [36.4–59.8] | 15 (75.0%) [50.9–91.3] | 0.031 |
|  | No | 39 (52.0%) [40.2–63.6] | 5 (25.0%) [8.7–49.1] |  |
|  | Don’t know | 0 | 0 |  |
| K9. HPV infection can occur even without symptoms | Yes | 40 (53.3%) [41.4–64.9] | 9 (45.0%) [23.1–68.5] | 0.508 |
|  | No | 35 (46.7%) [35.1–58.6] | 11 (55.0%) [31.5–76.9] |  |
|  | Don’t know | 0 | 0 |  |
| K10. Pap smear is a screening test for cervical cancer. | Yes | 37 (49.3%)[37.6–61.1] | 8 (40.0%) [19.1–63.9] | 0.458 |
|  | No | 38 (50.7%) [38.9–62.4] | 12 (60.0%) [36.1–80.9] |  |
|  | Don’t know | 0 | 0 |  |
| K11. Do you know where to get the HPV vaccine? | Yes | 40 (53.3%) [41.4–64.9] | 0 (0.0%) [0.0–16.8] | <0.001 |
|  | No | 35 (46.7%) [35.1–58.6] | 20 (100.0%) [83.2–100.0] |  |
