# Supplementary material for: Perceptions and practices among students, parents, and teachers of the Human Papillomavirus (HPV) Vaccination: A pilot feasibility study from Rural Kerala
Source: PLoS One. 2026 Aug 10;21(8):e0340559. doi: 10.1371/journal.pone.0340559 (PMC13456282; doi:10.1371/journal.pone.0340559)
Supplement: Supplementary file 2 [file pone.0340559.s002.docx]

S2 Table. Item-level comparison of attitudes towards HPV vaccination among students and teachers.

| Attitude Questions | Answer Options | Students (%)[C.I] | Teachers | p-value |
| --- | --- | --- | --- | --- |
| A1. HPV vaccination should be included in the national immunization schedule. | Strongly Agree | 15(20%)[11.8–31] | 2 (10.0%) [1.2–31.7] | 0.515 |
|  | Agree | 15(20%)[11.8–31] | 6 (30.0%) [11.9–54.3] |  |
|  | Neutral | 19 (25.3%) [16.0–37.1] | 4 (20.0%) [5.7–43.7] |  |
|  | Disagree | 15 (20.0%) [11.8–31.2] | 4 (20.0%) [5.7–43.7] |  |
|  | Strongly Disagree | 11 (14.7%) [7.6–24.7] | 4 (20.0%) [5.7–43.7] |  |
| A2. I believe HPV vaccination is important for cancer prevention. | Strongly Agree | 11 (14.7%) [7.6–24.7] | 2 (10.0%) [1.2–31.7] | 0.724 |
|  | Agree | 12(16.0%)[8.5-26.7] | 4 (20.0%) [5.7–43.7] |  |
|  | Neutral | 16 (21.3%) [12.8–32.2] | 3 (15.0%) [3.2–37.9] |  |
|  | Disagree | 15 (20.0%) [11.8–31.2] | 5 (25.0%) [8.7–49.1] |  |
|  | Strongly Disagree | 21 (28.0%) [18.3–39.6] | 6 (30.0%) [11.9–54.3] |  |
| A3. I would recommend the HPV vaccine to others. | Strongly Agree | 15 (20.0%) [11.8–31.2] | 8 (40.0%) [19.1–63.9] | 0.921 |
|  | Agree | 18 (24.0%) [14.9–35.3] | 4 (20.0%) [5.7–43.7] |  |
|  | Neutral | 10 (13.3%) [6.6–23.2] | 3 (15.0%) [3.2–37.9] |  |
|  | Disagree | 17 (22.7%) [13.8–34.5] | 2 (10.0%) [1.2–31.7] |  |
|  | Strongly Disagree | 14 (18.7%) [10.6–29.3] | 3 (15.0%) [3.2–37.9] |  |
| A4. I worry about the side effects of the HPV vaccine. | Strongly Agree | 19 (25.3%) [16.0–37.1] | 3 (15.0%) [3.2–37.9] | 0.378 |
|  | Agree | 14 (18.7%) [10.6–29.3] | 8 (40.0%) [19.1–63.9] |  |
|  | Neutral | 17 (22.7%) [13.8–34.5] | 3 (15.0%) [3.2–37.9] |  |
|  | Disagree | 10 (13.3%) [6.6–23.2] | 2 (10.0%) [1.2–31.7] |  |
|  | Strongly Disagree | 115 (20.0%) [11.8–31.2] | 4 (20.0%) [5.7–43.7] |  |
| A5. HPV vaccination promotes early sexual activity | Strongly Agree | 13 (17.3%) [9.5–28.0] | 4 (20.0%) [5.7–43.7] | 0.353 |
|  | Agree | 11 (14.7%) [7.6–24.7] | 2 (10.0%) [1.2–31.7] |  |
|  | Neutral | 20 (26.7%) [17.2–38.1] | 5 (25.0%) [8.7–49.1] |  |
|  | Disagree | 11(14.7%)[7.6-24.7] | 2 (10.0%) [1.2–31.7] |  |
|  | Strongly Disagree | 20 (26.7%) [17.2–38.1] | 7 (35.0%) [15.4–59.2] |  |
| A6. I believe awareness campaigns are needed for HPV vaccination. | Strongly Agree | 14 (18.7%) [10.6–29.3] | 1 (5.0%) [0.1–24.9] | 0.915 |
|  | Agree | 13 (17.3%) [9.5–28.0] | 3 (15.0%) [3.2–37.9] |  |
|  | Neutral | 17(22.7%)[13.8-34.5] | 8 (40.0%) [19.1–63.9] |  |
|  | Disagree | 13 (17.3%) [9.5–28.0] | 5 (25.0%) [8.7–49.1] |  |
|  | Strongly Disagree | 18 (24.0%) [14.9–35.3] | 3 (15.0%) [3.2–37.9] |  |
| A7. I trust the safety of the HPV vaccine. | Strongly Agree | 3 (15.0%) [3.2–37.9] | 1 (5.0%) [0.1–24.9] | 0.310 |
|  | Agree | 13 (17.3%) [9.5–28.0] | 3 (15.0%) [3.2–37.9] |  |
|  | Neutral | 17 (22.7%)[13.8-34.5] | 8 (40.0%) [19.1–63.9] |  |
|  | Disagree | 13 (17.3%) [9.5–28.0] | 5 (25.0%) [8.7–49.1] |  |
|  | Strongly Disagree | 18 (24.0%) [14.9–35.3] | 3 (15.0%) [3.2–37.9] |  |
| A8. If not vaccinated, are you willing to take it? | Yes | 33 (44.0%) [32.5–55.9] | 8 (40.0%) [19.1–63.9] | 0.874 |
|  | No | 42 (56.0%) [44.1–67.5] | 12 (60.0%) [36.1–80.9] |  |
