# Supplementary material for: Perceptions and practices among students, parents, and teachers of the Human Papillomavirus (HPV) Vaccination: A pilot feasibility study from Rural Kerala
Source: PLoS One. 2026 Aug 10;21(8):e0340559. doi: 10.1371/journal.pone.0340559 (PMC13456282; doi:10.1371/journal.pone.0340559)
Supplement: Supplementary file 3 [file pone.0340559.s003.docx]

S3 Table. Item-level comparison of HPV vaccination practices among students and teachers.

| Practice Questions | Answer Options | Students | Teachers | P value |
| --- | --- | --- | --- | --- |
| P1. Have you heard about the HPV vaccine before? | Yes | 45 (60.0%) [48.9–71.1] | 10 (50.0%) [27.2–72.8] | (p = 0.421) |
|  | No | 30 (40.0%) [28.9–51.1] | 10 (50.0%) [27.2–72.8] |  |
| P2. Have you ever received the HPV vaccine ? | Yes | 39 (52.0%) [40.2–63.6] | 39 (52.0%) [40.2–63.6] | (p = 0.874) |
|  | No | 36(48.0%)[36.4-59.8] | 10 (50.0%) [27.2–72.8] |  |
| P3. Have you encouraged anyone to take the HPV vaccine? | Yes | 30 (40.0%) [28.9–51.1] | 11 (55.0%) [31.5–76.9] | (p = 0.229) |
|  | No | 45 (60.0%) [48.9–71.1] | 9 (45.0%) [23.1–68.5] |  |
