# Supplementary material for: Perceptions and practices among students, parents, and teachers of the Human Papillomavirus (HPV) Vaccination: A pilot feasibility study from Rural Kerala
Source: PLoS One. 2026 Aug 10;21(8):e0340559. doi: 10.1371/journal.pone.0340559 (PMC13456282; doi:10.1371/journal.pone.0340559)
Supplement: Supplementary file 4 [file pone.0340559.s004.docx]

S4 Table. Summary of key findings and implications from the pilot feasibility study on HPV vaccination knowledge, attitudes, and practices.

| Domain | Observations | Implication |
| --- | --- | --- |
| Knowledge | ~50% knew HPV causes cervical cancer | Large gaps despite high literacy |
| Misconceptions | HPV affects only women; vaccine not available | Targeted correction needed |
| Attitudes | Neutral or uncertain responses dominant | Hesitancy rather than rejection |
| Safety Concerns | Common among both groups | Need for counselling |
| Uptake | Low vaccination despite willingness | Access and family involvement critical |
