# Supplementary material for: Perceptions and practices among students, parents, and teachers of the Human Papillomavirus (HPV) Vaccination: A pilot feasibility study from Rural Kerala
Source: PLoS One. 2026 Aug 10;21(8):e0340559. doi: 10.1371/journal.pone.0340559 (PMC13456282; doi:10.1371/journal.pone.0340559)
Supplement: Supplementary file 5 [file pone.0340559.s005.docx]

**S1 File** – Original questionnaire

Student Questionnaire

1. K1.HPV infection can cause cervical cancer
A. Yes
B. No
C. Don’t know
2. K2.HPV is a sexually  transmitted infection.

A. Yes

B. No

C. Don’t know

3. K3. HPV affects only women.
A. Yes
B. No
C. Don’t know
4. K4. The HPV vaccine is most effective when given before sexual debut
A. Yes
B. No
C. Don’t know
5. K5. The HPV vaccine is available in India.
A. Yes
B. No
C. Don’t know
6. K6. The HPV vaccine protects against all types of HPV.
A. Yes
B. No
C. Don’t know
7. K7. Boys can also receive the HPV vaccine.
A. Yes
B. No
C. Don’t know
8. K8. The HPV vaccine requires more than one dose.
A. Yes
B. No
C. Don’t know
9. K9. HPV infection can occur even without symptoms
A. Yes
B. No
C. Don’t know
10. K10. Pap smear is a screening test for cervical cancer.
A. Yes
B. No
C. Don’t know
11. K11. Do you know where to get the HPV vaccine?
A. Yes
B. No

Attitude

1. A1. HPV vaccination should be included in the National Immunization Schedule
A. Strongly Agree
B. Agree
C. Neutral
D. Disagree
E. Strongly Disagree .
2. A2. I believe HPV vaccination is important for cancer prevention.
A. Strongly Agree
B. Agree
C. Neutral
D. Disagree
E. Strongly Disagree
3. A3. I would recommend the HPV vaccine to others.
a. Strongly Agree
b. Agree
c. Neutral
d. Disagree
e. Strongly Disagree
4. A4. I worry about the side effects of the HPV vaccine.
A. Strongly Agree
B. Agree
C. Neutral
D. Disagree
E. Strongly Disagree
5. A5. HPV vaccination promotes early sexual activity.
A. Strongly Agree
B. Agree
C. Neutral
D. Disagree
E. Strongly Disagree
6. A6. I believe awareness campaigns are needed for the HPV vaccination.
A. Strongly Agree
B. Agree
C. Neutral
D. Disagree
E. Strongly Disagree
7. A7. I trust the safety of the HPV vaccine.
A. Strongly Agree
B. Agree
C. Neutral
D. Disagree
E. Strongly Disagree
8. A8. If you are not vaccinated, are you willing to take it ?
A. Strongly Agree
B. Agree
C. Neutral
D. Disagree
E. Strongly disagree

Practice

1. P1. Have you heard about the HPV vaccine before ?
A. Yes
B. No
2. P2. Have you received the HPV vaccine?
A. Yes
B. No
3. P3. Have you encouraged anyone to take the HPV vaccine?
A. Yes
B. No
4. P4. If not vaccinated, main reason:
A. Lack of awareness
B. Cost
C. Fear of side effects
D. Not advised by doctor
E. Cultural/ Religious reasons
5. P5. Would you be willing to take the HPV vaccine /vaccinate your daughter or son in the future ?
A. Yes
B. No
C. Not sure
