# Supplementary material for: Perceptions and practices among students, parents, and teachers of the Human Papillomavirus (HPV) Vaccination: A pilot feasibility study from Rural Kerala
Source: PLoS One. 2026 Aug 10;21(8):e0340559. doi: 10.1371/journal.pone.0340559 (PMC13456282; doi:10.1371/journal.pone.0340559)
Supplement: Supplementary file 6 [file pone.0340559.s006.docx]

**S2 File** – Revised questionnaire

# **SECTION A. SOCIODEMOGRAPHIC INFORMATION**

## **For Students**

1. Age (years)
2. Sex
   - Male
   - Female
   - Other
3. Class
   - 5–7
   - 8–10
   - 11–12
4. Religion
   - Hindu
   - Muslim
   - Christian
   - Other
5. Type of family
   - Nuclear
   - Joint
   - Extended
6. Father's education
   - No formal education
   - Primary
   - Secondary
   - Higher secondary
   - Graduate
   - Postgraduate
7. Mother's education
   - Same categories
8. Father's occupation
9. Mother's occupation
10. Who usually makes health-related decisions for you?

- Mother
- Father
- Both parents
- Grandparents
- Other

11. Have you ever attended a health education session on HPV or cervical cancer?

- Yes
- No

## **For Teachers**

1. Age
2. Sex
3. Subject taught
4. Years of teaching experience
5. Previous training on:
   - Adolescent health
   - HPV
   - Vaccination programmes
6. Have you ever participated in a school vaccination programme?
   - Yes
   - No

# **SECTION B. KNOWLEDGE**

### **Existing Questions**

K1–K11 (retain)

### **Additional Questions**

### **K12**

HPV infection can also affect males.

- Yes
- No
- Don't know

### **K13**

HPV infection can cause cancers other than cervical cancer.

- Yes
- No
- Don't know

### **K14**

The HPV vaccine can prevent most cervical cancers.

- Yes
- No
- Don't know

### **K15**

The HPV vaccine is recommended for adolescents aged 9–14 years.

- Yes
- No
- Don't know

### **K16**

The HPV vaccine is included in government vaccination programmes in some parts of India.

- Yes
- No
- Don't know

### **K17**

A person can still benefit from HPV vaccination even if they feel healthy.

- Yes
- No
- Don't know

### **K18**

HPV vaccination does not eliminate the need for cervical cancer screening later in life.

- Yes
- No
- Don't know

### **K19**

HPV infection usually clears without symptoms.

- Yes
- No
- Don't know

### **K20**

Healthcare providers recommend HPV vaccination.

- Yes
- No
- Don't know

# **SECTION C. ATTITUDES**

Retain A1–A8.

New Questions

### **A9**

I feel I have enough information to make a decision about HPV vaccination.

### **A10**

My parents/family would support HPV vaccination.

### **A11**

I trust information provided by healthcare professionals about HPV vaccination.

### **A12**

I trust information about HPV vaccination provided through social media.

### **A13**

HPV vaccination should be offered through schools.

### **A14**

I would be more likely to receive HPV vaccination if recommended by a doctor.

### **A15**

I would be more likely to receive HPV vaccination if it were provided free of cost.

### **A16**

I feel comfortable discussing HPV vaccination with parents or teachers.

(All measured using 5-point Likert scale)

# **SECTION D. PRACTICES**

Retain P1–P5.

Modifying P2:

### **P2. Have you received the HPV vaccine?**

- Yes, completed all doses
- Yes, partially vaccinated
- No
- Don't know

Add:

### **P6.** Have you ever discussed HPV vaccination with:

| **Person** | **Yes** | **No** |
| --- | --- | --- |
| Parent | □ | □ |
| Teacher | □ | □ |
| Doctor | □ | □ |
| Friend | □ | □ |

### **P7.** Have you ever searched for information about HPV vaccination?

- Yes
- No

### **P8.** Have you ever attended a health awareness programme on HPV vaccination?

- Yes
- No

**SECTION E. SOURCES OF INFORMATION**(Identified as a major gap in the pilot)

### **E1.** Where did you first hear about HPV vaccination?

(Multiple responses allowed)

- School
- Teacher
- Doctor
- Nurse
- Parent
- Friend
- Television
- Newspaper
- Social media
- Internet
- Other

### **E2.** Which source do you trust most?

- Doctor
- Nurse
- Teacher
- Parent
- Government
- Social media
- Religious leader
- Friends

# **SECTION F. BARRIERS TO HPV VACCINATION**

This was the biggest weakness identified in the pilot.

### **If not vaccinated,** what is the main reason?

(Multiple responses allowed)

- I have never heard of the vaccine
- I do not know where it is available
- Cost
- Fear of side effects
- Parents do not agree
- Family members do not agree
- Religious concerns
- Cultural concerns
- Fear of injections
- Doctor has not recommended it
- School has not provided information
- Vaccine not easily available
- Too young
- Not necessary
- Other ________

# **SECTION G. PARENTAL AND HOUSEHOLD DECISION-MAKING**

(New section based on pilot findings)

### **G1.** Who would make the final decision regarding HPV vaccination?

- Mother
- Father
- Both parents
- Student
- Grandparents
- Other

### **G2.** Before deciding about HPV vaccination, whose opinion would your family seek?(Multiple responses)

- Doctor
- Teacher
- Religious leader
- Family member
- Community leader
- Internet/social media

### **G3.** Would your family be willing to discuss HPV vaccination with a healthcare professional?

- Yes
- No
- Not sure

# **SECTION H. TEACHER PREPAREDNESS (TEACHERS ONLY)**

### **T1.** Do you feel confident explaining HPV vaccination to students?

- Yes
- No
- Not sure

### **T2.** Would you be willing to participate in a school HPV vaccination programme?

- Yes
- No
- Not sure

### **T3.** Do you feel you need additional training on HPV and HPV vaccination?

- Yes
- No

### **T4.** Would you support school-based HPV awareness sessions?

- Yes
- No
- Not sure
