# Supplementary material for: Perceptions and practices among students, parents, and teachers of the Human Papillomavirus (HPV) Vaccination: A pilot feasibility study from Rural Kerala
Source: PLoS One. 2026 Aug 10;21(8):e0340559. doi: 10.1371/journal.pone.0340559 (PMC13456282; doi:10.1371/journal.pone.0340559)
Supplement: Supplementary file 7 [file pone.0340559.s009.docx]

| **Date:** | June 3, 2026 |
| --- | --- |
| **From** | "Anjum John" dranjumsjohn@pimsrc.edu.in;johnanj@gmail.com |
| **To :** | "PLOS ONE" plosone@plos.org |
| **Subject:** | PLOS ONE Decision: Revision required [PONE-D-25-67419] |

PONE-D-25-67419

Perceptions and Practices Among Students, Parents, and Teachers of the Human Papilloma Virus( HPV) vaccine : A Pilot Feasibility Study from Rural Kerala

Dear Editors and Reviewers,

Thank you for reviewing our manuscript in such detail and your efforts to make the manuscript as descriptive and informative as possible for readers. Thank you for the merit you see in this pilot study and encouragement to publish a pilot study with its methodological flaws reported in its completeness. We agree that our work was not perfect, and we have learned from this experience. Accordingly, we are submitting a revised version of the manuscript that addresses the points raised during the review process. Thank you for accommodating our request for extensions of the deadline for submission of the revised manuscript.

We are including the following:

1. A letter that responds to each point raised by the academic editor and reviewer(s). This letter is uploaded as a separate file labelled “ Response to Reviewers”.
2. A marked-up copy of your manuscript that highlights changes made to the original version. This is uploaded as a separate file labeled 'Revised Manuscript with Track Changes'.
3. An unmarked version of the revised paper without tracked changes. This is uploaded as a separate file labeled 'Manuscript'.

No changes have been made to our financial disclosure, and hence no statement has been updated in our cover letter. Please do note that we have no funding for this research or publication as our institution does not support any such funding.
Kind regards,

Anjum John, Corresponding author

**Specific comments and replies**

Journal Requirements:

When submitting your revision, we need you to address these additional requirements.

1. Please ensure that your manuscript meets PLOS ONE's style requirements, including those for file naming. The PLOS ONE style templates can be found at https://journals.plos.org/plosone/s/file?id=wjVg/PLOSOne_formatting_sample_main_body.pdf and <https://journals.plos.org/plosone/s/file?id=ba62/PLOSOne_formatting_sample_title_authors_affiliations.pdf>

We have done our best to ensure that our manuscript meets PLOS One’s style requirements.

2. Please include your full ethics statement in the ‘Methods’ section of your manuscript file. In your statement, please include the full name of the IRB or ethics committee who approved or waived your study, as well as whether or not you obtained informed written or verbal consent. If consent was waived for your study, please include this information in your statement as well.

Our full ethics statement has been included in the “Methods” section of the manuscript file. The full name of the IRB, which approved the study and details about the informed consent have been included.

3. In the online submission form, you indicated that the data cannot be shared publicly because of privacy reasons but they are available with the authors and will be provided anonymously, upon reasonable request. All PLOS journals now require all data underlying the findings described in their manuscript to be freely available to other researchers, either 1. In a public repository, 2. Within the manuscript itself, or 3. Uploaded as supplementary information.
This policy applies to all data except where public deposition would breach compliance with the protocol approved by your research ethics board. If your data cannot be made publicly available for ethical or legal reasons (e.g., public availability would compromise patient privacy), please explain your reasons on resubmission and your exemption request will be escalated for approval.

Response : Thank you for highlighting the journal's data availability requirements.

The dataset underlying this pilot study contains individual-level survey responses collected from school-going adolescents aged 11–15 years and teachers. Although direct identifiers were removed during data entry, the combination of demographic and response variables from a single-school pilot study conducted in a small rural setting may create a risk of indirect participant identification. Participant confidentiality therefore remains an important ethical consideration.

To comply with the journal's data-sharing policy while protecting participant privacy, we have prepared a fully anonymized dataset with all direct identifiers removed and with potentially identifying variables appropriately minimized. This anonymized dataset will be provided as Supporting Information accompanying the manuscript. No information that could reasonably be used to identify individual participants has been included.

We have revised the Data Availability Statement accordingly and trust that this approach satisfies the journal's requirements for transparency and reproducibility while maintaining participant confidentiality.

4. When completing the data availability statement of the submission form, you indicated that you will make your data available on acceptance. We strongly recommend all authors decide on a data sharing plan before acceptance, as the process can be lengthy and hold up publication timelines. Please note that, though access restrictions are acceptable now, your entire data will need to be made freely accessible if your manuscript is accepted for publication. This policy applies to all data except where public deposition would breach compliance with the protocol approved by your research ethics board. If you are unable to adhere to our open data policy, please kindly revise your statement to explain your reasoning and we will seek the editor's input on an exemption. Please be assured that, once you have provided your new statement, the assessment of your exemption will not hold up the peer review process.

Response to Comment 4:

Thank you for this clarification. We agree with the journal's recommendation to establish a data-sharing plan prior to acceptance. To comply with the journal's Open Data Policy, we have revised our Data Availability Statement. A fully anonymized dataset underlying the findings reported in this manuscript will be provided as Supporting Information and will be freely accessible upon publication. All direct identifiers have been removed, and the dataset has been carefully reviewed to minimize the risk of participant identification while preserving the integrity of the analyses.

We believe this approach balances transparency and reproducibility with our ethical obligation to protect participant confidentiality, particularly given that the study involved adolescent school students. The revised Data Availability Statement has been updated accordingly in the manuscript and submission system.

5. We note you have included a table to which you do not refer in the text of your manuscript. Please ensure that you refer to Table 1, 2, 3 in your text; if accepted, production will need this reference to link the reader to the Table.

Response to Comment 5:Thank you for bringing this to our attention. The table summarizing the key findings at the end of the manuscript was not explicitly cited in the text. To address this issue and avoid redundancy with the main results tables, we have moved this summary table to the Supporting Information as Supporting Information S4 Table.( Summary of key findings and implications from the pilot feasibility study on HPV vaccination knowledge, attitudes, and practices.) All remaining tables are now cited sequentially within the main text.

6. Please include captions for your Supporting Information files at the end of your manuscript, and update any in-text citations to match accordingly. Please see our Supporting Information guidelines for more information: <http://journals.plos.org/plosone/s/supporting-information>

Response : Captions for Supporting Information files have been included to the end of the manuscript and in-text citations have been updated to match, with reference to the Supporting Information Guidelines of the journal.

7. [Please remove all personal information, ensure that the data shared are in accordance with participant consent, and re-upload a fully anonymized data set.](http://www.bmj.com/content/340/bmj.c181.long.)

Response : The shared data set has all personal information removed and data is shared in accordance with participant consent, and a fully anonymized dataset has been re-uploaded. Spreadsheet columns with personal information have been removed and not hidden as directed.  [Additional guidance on preparing raw data for publication can be found in our Data Policy (https://journals.plos.org/plosone/s/data-availability#loc-human-research-participant-data-and-other-sensitive-data) and in the following article: http://www.bmj.com/content/340/bmj.c181.long.](http://www.bmj.com/content/340/bmj.c181.long.)

8. If the reviewer comments include a recommendation to cite specific previously published works, please review and evaluate these publications to determine whether they are relevant and should be cited. There is no requirement to cite these works unless the editor has indicated otherwise.

Response : Thank you.

**Additional Editor Comments :

Manuscript ID: PONE-D-25-67419
Title: Perceptions and Practices Among Students, Parents, and Teachers of the Human Papilloma Virus (HPV) Vaccine: A Pilot Feasibility Study from Rural Kerala
Corresponding Author: Anjum John, MD, MPH
Date of Review: January 10, 2026

Overall Assessment**
This manuscript presents a pilot feasibility study exploring HPV vaccine-related knowledge, attitudes, and practices (KAP) among students, teachers, and parents in rural Kerala. The topic is of high public health relevance, particularly in light of India’s planned national HPV vaccine rollout. The study is framed appropriately as a pilot, with clear feasibility objectives. However, the manuscript suffers from significant methodological and ethical shortcomings, incomplete reporting, and overinterpretation of limited data. In its current form, it does not meet the standards for publication in PLOS ONE.
Decision: Major Revision Required
Detailed Review by Section
1. Title and Abstract
• Title: Clear but contains a typographical error (“Human Papilloma Virus (HPV)”). Correct spacing and punctuation are needed.

Response : We thank the reviewer for these valuable observations. We have corrected the title to read “Human Papillomavirus (HPV)” throughout the manuscript.
• Abstract:
o Appropriately summarizes the pilot’s purpose and main findings.
o However, it fails to mention the critical limitation of zero parent participation, which fundamentally affects the study’s feasibility conclusions.

o The statement “multivariable analysis was not undertaken” is misleading, it implies it was considered but omitted due to data, rather than being inherently outside the scope of a pilot.
o Recommendation: Explicitly state the absence of parent data and clarify that the pilot’s goal was tool/process refinement, not hypothesis testing.

Response : We thank the reviewer for these valuable observations. We agree that the absence of parent participation is an important finding from a feasibility perspective and have now explicitly reported this limitation in the Abstract. The Abstract has been revised to explicitly report the absence of parent participation, clarify the feasibility-focused objectives of the pilot study, and remove the implication that multivariable analyses were planned but omitted. The Conclusions have also been revised to emphasize methodological insights gained from the pilot.

2. Introduction
• Provides a comprehensive background on HPV, cervical cancer burden, and vaccination challenges in India and Kerala.
• Literature review is adequate, though somewhat dense. Could be streamlined to focus more on the KAP evidence gap in school settings.
• Rationale is clearly stated: to pilot a questionnaire and assess feasibility for a larger study.

Response:

We thank the reviewer for the positive assessment of the Introduction and rationale. In response to the suggestion, we have streamlined portions of the literature review to reduce background detail and place greater emphasis on the existing evidence gaps regarding HPV-related knowledge, attitudes, and practices among school-based stakeholders, particularly students, parents, and teachers. The rationale for the pilot study has also been further clarified to emphasize its feasibility and questionnaire-development objectives.
3. Methods
3.1. Study Design and Setting
• Appropriately described as a pilot cross-sectional study.
• However, study dates are inconsistent: data collection is noted as June 2025 in the methods, but submission is dated December 2025. This suggests either prospective dating or a clerical error that needs correction.

Comment : We thank the reviewer for this observation. We have carefully reviewed the manuscript and confirmed that data collection was conducted between 18 and 19 June 2025, as stated in the Methods section. The manuscript was subsequently prepared and submitted after completion of data collection and analysis. We were unable to identify any conflicting study dates within the manuscript text and believe the reviewer may be referring to the manuscript submission date recorded in the journal system. To avoid any ambiguity, we have reviewed and clarified all study dates in the revised manuscript.
3.2. Ethics
• Ethical approval is noted, but the consent process is problematic.
o Parental consent was sought but not obtained because “parents were unavailable.” This is a major flaw; parental participation was a key study aim. The researchers should have implemented a follow-up mechanism (e.g., phone calls, rescheduled meetings).
o Verbal assent from students is acceptable, but details of how assent was documented are missing.

Comment : We thank the reviewer for this important observation. We acknowledge that the absence of parental participation represents a significant limitation of the study. However, as this investigation was designed as a pilot feasibility study, one of its objectives was to identify practical challenges associated with recruitment and study implementation. The inability to obtain parent participation despite distributing information sheets and questionnaires through students highlighted an important feasibility barrier that will inform the design of the larger study.

We agree that additional recruitment strategies such as follow-up telephone calls, parent meetings, reminder notices, or rescheduled data collection sessions could improve parental participation. These approaches have now been acknowledged in the Discussion and incorporated into the proposed modifications for the full-scale study.

Regarding student assent, verbal assent was obtained from all participating students after providing age-appropriate information about the study. Investigators maintained a study log documenting assent procedures and participant enrolment. Additional details regarding the assent process have now been added to the Methods section for clarity.

3.3. Questionnaire Development
• Describes content validation, pilot testing, and Cronbach’s alpha, which is good.
• However, the questionnaire itself is not provided in the submission (neither in main text nor supplementary). This is essential for reproducibility.

Response: We thank the reviewer for this valuable suggestion. We agree that providing the questionnaire enhances transparency, reproducibility, and the utility of the study for future researchers. Accordingly, the complete questionnaire used in this pilot study has now been included as Supporting Information (Supplementary File S1). Appropriate references to the supplementary questionnaire have been added in the Methods section.
• Cronbach’s alpha is reported as ≥0.7, but no actual value or breakdown by subscale (knowledge, attitude, practice) is given.

Response : Thank you for this observation. We agree that reporting only a threshold value was insufficient. We have revised the manuscript to provide the actual Cronbach's alpha coefficients obtained during preliminary pilot testing of the questionnaire. The overall questionnaire demonstrated acceptable internal consistency (Cronbach's α = 0.73). Domain-specific alpha coefficients were 0.63 for the knowledge domain, 0.65 for the attitude domain, and 0.24 for the practice domain. As these estimates were derived from a small pilot-testing sample and the primary objective was questionnaire refinement rather than psychometric validation, the reliability analyses were used to identify areas requiring further improvement before implementation of the main pilot feasibility study. The relatively low internal consistency observed for the practice domain likely reflects the heterogeneous nature of vaccination-related behaviours and further supports the need for refinement of these items in future studies.
3.4. Data Collection
• Paper-based forms were used, but no information on data quality checks or inter-rater reliability is provided.

Response : We thank the reviewer for this observation. As the questionnaire was self-administered and responses were recorded directly by participants, inter-rater reliability was not applicable to this study. However, we agree that data quality procedures should be described more clearly. We have revised the Methods section to state that completed questionnaires were reviewed for completeness before data entry, data were entered into password-protected electronic spreadsheets, and entries were cross-checked for accuracy prior to analysis.
• The statement that “no sociodemographic information was collected” is a major methodological weakness for a KAP study, even as a pilot. Socioeconomic and educational factors are key predictors of vaccine awareness and uptake.

Response : We agree with the reviewer that sociodemographic and contextual variables are important determinants of HPV vaccine knowledge, attitudes, and practices. The omission of these variables represents an important limitation of the pilot study. However, one of the objectives of the pilot was to identify deficiencies in the questionnaire and determine which variables should be incorporated into the full-scale study. The pilot findings highlighted the inability to explore predictors of HPV vaccine-related outcomes without information on socioeconomic, educational, household, and information-exposure characteristics. We have strengthened the Discussion and Limitations sections to acknowledge this issue and have outlined the specific sociodemographic and contextual variables that will be included in the larger study. We have also included a modified questionnaire including all details that should be included based on the results of the pilot study. (**S2 File** – Revised questionnaire)

4. Results
4.1. Participant Flow
• Zero parent participation is a critical finding that should dominate the results and discussion of feasibility. It is currently underemphasized.

Comment : We thank the reviewer for these important observations. We agree that the absence of parental participation represents a major feasibility finding rather than merely a study limitation. The Results and Discussion sections have been revised to emphasize this issue and its implications for the design of the planned full-scale study. Additional discussion has been included regarding alternative parent-recruitment strategies that may improve participation in future research.

• Student and teacher demographics are provided, but the gender imbalance (78.7% female students, 90% female teachers) limits generalizability and may bias attitude responses.

Comments : We thank the reviewer for this observation. We acknowledge that the study population was predominantly female among both students and teachers, which may influence the generalizability of the findings and potentially affect HPV vaccine-related knowledge and attitude responses. This limitation has now been explicitly acknowledged in the Discussion and Limitations sections.

However, it is also important to note that Kerala has the highest sex ratio among Indian states, with 1,084 females per 1,000 males according to Census data, reflecting a naturally higher proportion of females in the population. Nevertheless, the female predominance observed in our sample exceeded that expected from the state population structure and may partly reflect the characteristics of the participating school and teaching workforce. Future studies will seek to recruit more gender-balanced samples to improve representativeness.

4.2. KAP Findings
• Tables 2-4 are clear and useful.
• However, results are presented without measures of uncertainty (no confidence intervals, no tests of difference between students and teachers).
• The authors note “no inferential analysis,” but even descriptive statistics should include percentages with denominators clearly stated (some totals in tables do not sum correctly).

Response: We thank the reviewer for these constructive comments. We have carefully reviewed and recalculated all frequencies and percentages directly from the original dataset and corrected inconsistencies identified in the tables. Denominators have now been clearly specified for students (n = 75) and teachers (n = 20), and percentages are reported accordingly.

To address the reviewer's suggestion, we have added 95% confidence intervals for the overall knowledge, attitude, and practice domain scores in the Results section. In addition, exploratory item-level comparisons between students and teachers, including confidence intervals and p-values, are now provided in Supplementary Table S2. These analyses are presented for completeness and are interpreted cautiously given the pilot nature of the study and limited sample size, given the primary purpose of this pilot study was feasibility assessment rather than hypothesis testing. No statistically significant differences were observed between the groups for knowledge (p = 0.169), attitude (p = 0.253), or practice (p = 0.568) domain scores. These findings have been interpreted cautiously given the pilot nature of the study and the limited sample size, particularly among teachers. The manuscript has been revised accordingly.

S1 Table. Item-level comparison of HPV-related knowledge among students and teachers

S2 Table. Item-level comparison of attitudes towards HPV vaccination among students and teachers.

S3 Table. Item-level comparison of HPV vaccination practices among students and teachers.

4.3. “Significant Misconceptions” Section
• This is interpretive and belongs in the Discussion, not Results.
• Phrases like “poor understanding,” “major gaps,” and “low-to-moderate” are subjective without benchmarking against validated thresholds.

Response: We thank the reviewer for this important observation. We agree that several statements in the Results section were interpretive rather than purely descriptive. Accordingly, we have revised the Results section to focus on the presentation of observed frequencies and percentages and have moved interpretive statements regarding misconceptions, knowledge gaps, and implications to the Discussion section. We have also removed subjective descriptors such as “poor understanding,” “major gaps,” and “low-to-moderate knowledge,” as no validated thresholds were available for categorizing these outcomes.
5. Discussion
• Overinterprets pilot data as if from a full-scale analytical study.
• Compares findings to national and global literature appropriately, but fails to contextualize the pilot’s severe limitations (single school, no parents, no sociodemographic).
• The section on “Proposed Changes to Questionnaire” is useful and appropriate for a pilot, but it is buried in the Discussion. It should be moved to a dedicated “Lessons Learned for Future Research” section.
6. Public Health Implications and Strengths/Limitations
• Strengths: Correctly identifies feasibility focus and tool validation.
• Limitations: Understates the impact of missing parent data and lack of sociodemographic variables.
• The limitation regarding “self-reported practices” is noted, but not the social desirability bias inherent in vaccine attitude surveys in school settings.

Response: We thank the reviewer for these valuable observations. We agree that the primary purpose of this study was to assess feasibility, recruitment processes, and questionnaire performance rather than to generate generalizable estimates of HPV-related knowledge, attitudes, and practices. Accordingly, the Discussion has been revised to moderate interpretations of the findings and to place greater emphasis on the pilot nature of the study.

Additional text has been incorporated to explicitly acknowledge the implications of conducting the study in a single school, the complete absence of parental participation, and the lack of sociodemographic and contextual variables. We have also reorganized the manuscript by moving the section previously titled “Proposed Changes to Questionnaire” into a new subsection entitled “Lessons Learned for Future Research,” which highlights the methodological insights gained from the pilot and the modifications planned for the larger study.

The Limitations section has also been strengthened to discuss the potential influence of social desirability bias in self-reported attitudes and practices, particularly in a school-based setting.

7. Ethical and Reporting Compliance
• Ethics Statement: Provided, but the consent lapse with parents must be explicitly addressed as a limitation and a lesson learned.
• Data Availability: States data are “available upon request” due to privacy, which does not comply with PLOS ONE’s data policy. De-identified data must be deposited in a public repository or provided as supplementary files.
• Funding: Correctly declared as none.
8. Presentation and Language
• Writing: Generally clear but contains grammatical errors and informal phrasing (e.g., “vaccine shyness”).
• Tables: Well-structured but some formatting inconsistencies (e.g., missing totals, alignment).
• References: Extensive but some formatting issues (e.g., inconsistent journal abbreviations).

Major Revisions Required
1. Address Parent Non-Participation: This must be framed as a central feasibility finding, not just a deviation. Discuss why it happened and how it will be rectified in the main study.
2. Provide the Questionnaire: Include the full questionnaire as a supplementary file.
3. Deposit Data: Share de-identified data in a public repository or as supporting information.
4. Clarify Study Dates: Correct the temporal discrepancy between data collection (June 2025) and submission (December 2025).
5. Strengthen Methods: Describe how assent was documented and detail data quality control procedures.
6. Restructure Discussion: Separate “lessons learned for tool refinement” from interpretive comparison with other studies. Avoid overgeneralization.

Minor Revisions
1. Correct title punctuation: “Human Papillomavirus (HPV) Vaccine”.
2. Report exact Cronbach’s alpha value and subscale reliabilities.
3. Add confidence intervals or inter-group comparisons where appropriate.
4. Move “Significant Misconceptions” from Results to Discussion.
5. Proofread for language consistency and academic tone.

Final Comments
This pilot study addresses an important topic and appropriately focuses on feasibility and tool refinement. However, the manuscript in its current form lacks the methodological rigor, ethical completeness, and data transparency required for publication in PLOS ONE. With substantial revisions, particularly regarding parent participation, data sharing, and questionnaire transparency, the study could provide a valuable foundation for future research.
I recommend major revision and look forward to reviewing a revised version that addresses these critical concerns.
Signed,
Prof. Morufu Olalekan Raimi


[Note: HTML markup is below. Please do not edit.]


[NOTE: If reviewer comments were submitted as an attachment file, they will be attached to this email and accessible via the submission site. Please log into your account, locate the manuscript record, and check for the action link "View Attachments". If this link does not appear, there are no attachment files.]

To ensure your figures meet our technical requirements, please review our figure guidelines: https://journals.plos.org/plosone/s/figures

You may also use PLOS’s free figure tool, NAAS, to help you prepare publication quality figures: https://journals.plos.org/plosone/s/figures#loc-tools-for-figure-preparation.

NAAS will assess whether your figures meet our technical requirements by comparing each figure against our figure specifications.

Xxxxxxxxxxxxxxxxxxxxxxxxxxxxxxxxxxxxxxxxxxxxxxxxxxxxxxxxxxxxxxxxxxxxxxxxxxxxxxxxxxxxxxxxxxxxx

**Title** : **Perceptions and Practices Among Students, Parents, and Teachers of the Human Papilloma Virus( HPV) vaccine : A Pilot Feasibility Study from Rural Kerala**

**Short Title:** HPV Vaccine KAP in a Kerala School

**Authors :** Anjum John^1^, Roshna Rasheed^1^, Mercy John Idikula^1^

**Institutional Affiliation** : Pushpagiri Institute of Medical Sciences & Research Center, Tiruvalla( city), Kerala( State), India ( Country)

**Contact Details** : Dr. Anjum John, Assistant Professor, Community Medicine, Pushpagiri Institute of Medical Sciences & Research Center, Tiruvalla( city), Kerala( State), India ( Country)

Dr. Roshna Rasheed, Junior Resident, Community Medicine, Pushpagiri Institute of Medical Sciences & Research Center, Tiruvalla( city), Kerala( State), India ( Country)

Dr. Mercy John Idikula, Professor and Head of the Department of Microbiology, Pushpagiri Institute of Medical Sciences & Research Center, Tiruvalla( city), Kerala( State), India ( Country)

**Corresponding Author** : Dr. Anjum John, Assistant Professor, Community Medicine, Pushpagiri Institute of Medical Sciences & Research Center, Tiruvalla( city), Kerala( State), India ( Country)

Phone number : 0091 7510714994

Email : dranjumsjohn@pimsrc.edu.in

**Funding Declaration** : There has been no funding for the manuscript.

**Clinical Trial Number** : Not applicable

**Consent to Publish Declaration** : Not applicable

**Perceptions and Practices Among Students, Parents, and Teachers of the Human Papilloma Virus( HPV) vaccine : A Pilot Feasibility Study from Rural Kerala**

**Abstract**

B**ackground:** India bears the world’s highest cervical cancer burden, yet awareness and uptake of the effective HPV vaccine remain low. Although Kerala has initiated pilot HPV vaccination programmes, stakeholder knowledge, attitudes, and practices (KAP) are not well documented. This pilot study aimed to assess the feasibility of a KAP study and to identify gaps in the questionnaire, for future analytical modelling.

**Methods:** A cross-sectional pilot study was conducted in a school in Pulinkunnu, Kerala, among students aged 11–15 years, their parents, and teachers. A structured, validated questionnaire, developed in English, translated into Malayalam, was administered during school hours. Responses were entered into secure electronic spreadsheets. Descriptive statistics summarized KAP outcomes. As key demographic and behavioral predictors—such as gender, socioeconomic status, prior health education, and exposure to HPV information—were not captured, multivariable analysis was not undertaken.

**Results:** The pilot included 75 students and 20 teachers, predominantly female. HPV-related knowledge was low across groups, with widespread misconceptions and limited awareness of vaccine availability and cervical cancer screening. Attitudes toward vaccination were mixed, marked by safety concerns and modest willingness to receive or recommend the vaccine. Practices reflected low prior awareness and uptake, with few respondents having heard of or received the HPV vaccine.

**Conclusions:** This pilot revealed major gaps in HPV-related knowledge, attitudes, and practices, among stakeholders and highlighted the need for an improved questionnaire in item clarity, response patterns, and missing variables and a larger study to identify predictors and inform targeted school-based HPV education efforts.

**Key Words**

Human Papilloma Virus, Vaccine, HPV, Cervical Cancer, School Students, KAP study, pilot study

Abstract Word Count : 248 words

**Ethics and Dissemination**

Ethical approval for this pilot study was obtained from the Institutional Ethics Committee of Pushpagiri Institute of Medical Sciences and Research Centre. Written informed consent was obtained from participating teachers, and parental consent was sought for students; however, parents were unavailable on the day of data collection, resulting in their non-participation. Verbal assent was obtained from all student participants prior to survey administration.

**Patient and Public Involvement**

Students, teachers, and parents were not involved in the development of the research protocol or the pilot questionnaire. As this was a preliminary feasibility study, no direct involvement in interpreting or disseminating the results is planned at this stage. Insights from the pilot will instead be used internally by the research team to refine the study tools and procedures for the full-scale investigation.

**Perceptions and Practices Among Students, Parents, and Teachers of the Human Papilloma Virus( HPV) vaccine : A Pilot Feasibility Study from Rural Kerala**

**Introduction**
 Human Papillomavirus (HPV) is the most common sexually transmitted infection globally, with around 40 strains associated with infections of the anogenital region. Although most of these infections are self-limiting, certain high-risk strains are linked to cancers such as cervical, anal, oropharyngeal, and penile cancer. (1)Despite the availability of effective vaccines, HPV-related diseases remain a major public health concern, particularly in low- and middle-income countries where screening and vaccination coverage are limited. Understanding public perspectives and practices (KAP) regarding HPV and its vaccine is therefore critical to shaping effective prevention strategies.(2-5)

**Public health burden of HPV**
 Globally, cervical cancer is the fourth most common cancer among women. In 2023, it was estimated that there were 604,000 new cervical cancer cases, causing around 342,000 deaths worldwide. Projections suggest an annual increase of nearly 100,000 new cases between 2018 and 2030. Prevalence of HPV is influenced by social and economic determinants like gender inequality and poverty, with illiteracy and early sexual initiation contributing to over 85% of cervical cancer cases, with an additional risk in people living with Human Immunodeficiency virus ( HIV) infections.(6, 7) The disease threatens the well-being of children when mothers die prematurely due to cervical cancer.(8)

**Disparities in healthcare**
 Cervical cancer is a preventable and curable disease. Despite the availability of the HPV vaccine for the prevention of cervical cancer , and effective treatment measures- much of the world has limited access to these services. Deaths due to cervical cancer are three times higher in low- or lower- middle- income countries, demonstrating the widespread disparities in the availability, accessibility, and adaptability of health care services in regions with the highest disease burden.(9)

Integrating HPV vaccination, cervical cancer screening, and management of precancerous lesions/cervical cancer into health systems remain essential. Until then, cervical cancer will continue to be one of the leading causes of cancer death in women. Involving people in their own healthcare helps reduce the incidence, mortality, and burden of HPV-related conditions. (10)The demonstrated success of HPV vaccination has prompted a global commitment to eliminate cervical cancer as a public health problem. (11)

**Global strategy**
 Public health microbiology is at the threshold of a rare but doable feat- the elimination of a non-communicable disease- cervical cancer. The World Health Organization’s 2020 Global Strategy to eliminate cervical cancer set the 90-70-90 targets by 2030: vaccinate 90% of girls under 15 years, screen 70% of women at 35 and 45 years of age, and treat 90% of women identified with cervical disease. (7, 11)

**HPV in India**
 India has the highest global burden of cervical cancer. Annually, about 132,000 new cases and 80,000 deaths occur, with a high prevalence of HPV type 16.(5, 6)The vastness of the country and the diversity of its population are responsible for the lack of proper documentation of the epidemiology and patterns of HPV virus strains. (10) Thus, as in other lower income countries, the implementation of prevention and control programs has been difficult. This highlights the importance of HPV vaccination in India.

**Vaccine coverage in India and Kerala**
 India licensed two HPV vaccines in 2008. The state of Sikkim successfully implemented the first school-based immunization program in 2018, achieving >95% coverage with strong government and community support. (12)In 2022, the National Technical Advisory Committee on Immunization recommended introducing the indigenously developed quadrivalent vaccine into the Universal Immunization Program (UIP). The vaccine rollout was planned for 2024, with Kerala’s Wayanad and Alappuzha districts included in the pilot phase.(13-16)

In Kerala, voluntary uptake remains poorly documented, and some studies report declining cervical cancer rates (7–9/100,000 women annually), which has sometimes been used to argue for screening rather than vaccination. (17) A 2018 study found only 8% coverage among medical students. (18) More recently, the Happy Noolpuzha Initiative in Wayanad demonstrated local-level success by vaccinating tribal girls through a Panchayat-led campaign.(19)

**Review of literature** Prior studies in India show low levels of HPV awareness and vaccination. Backer et al. (2024) found that although 85.6% of college students in Kerala knew HPV causes cervical cancer, only 48.6% had heard of the vaccine, and uptake was low (11.6% in medical students, 4.9% in non-medical).(20) Basu et al. similarly reported limited uptake among adolescents in North India, citing lack of awareness, cost, and cultural hesitancy. (21)School-based vaccination and awareness campaigns have proven effective in increasing acceptance.(22-24) However, teachers and parents often lack adequate knowledge, with safety concerns and stigma acting as major barriers. (25-27) Our full-scale study aimed to fill this gap by assessing stakeholder knowledge and perceptions in the Kerala context.

**Rationale for the present study**
 Limited evidence exists on the knowledge, attitudes, and practices of school-going adolescents, parents, and teachers—key stakeholders in vaccine acceptance. This gap limits the development of effective, school-based prevention strategies, despite ongoing HPV vaccination initiatives. Additionally, no validated tools were available to assess HPV-related knowledge and perceptions in this setting, necessitating the development and pilot testing of a new questionnaire. This pilot study was therefore undertaken to evaluate the feasibility of the tool, study item clarity and completeness of variables , and ensure that the instrument could adequately capture stakeholder perspectives before implementing a full-scale study. By identifying gaps in awareness, acceptance, and questionnaire performance, this pilot aimed to generate preliminary insights to guide a larger, more comprehensive investigation. The protocol for this study was published in the Indian Journal of Applied Research in October 2025.( doi: 10.36106/ijar and ISSN No : 2249-555X available from (<https://www.worldwidejournals.com/indian-journal-of-applied-research-(IJAR)/fileview/knowledge-attitude-and-practice-regarding-human-papillomavirus-hpv-vaccine-among-school-students-parents-and-teachers-in-central-kerala-a-crosssectional-study-protocol_October_2025_5111739822_7103697.pdf>)

**Objectives**

The study was conducted to assess the feasibility of measuring knowledge, attitudes, and practices related to HPV and HPV vaccination among school students aged 11–15 years, their teachers, and their parents, in preparation for a larger study.

**Methods**

**Study design and setting**

A pilot cross-sectional study was conducted in 2025 at a higher secondary school in Pulinkunnu, Alappuzha, Kerala, targeting students aged 11–15 years, their teachers, and their parents. This study was explicitly designed as a pilot to refine instruments, assess feasibility, and inform changes to the questionnaire and resource scale up, consistent with recommended guidelines for pilot and feasibility studies. (28, 29)

**Study participants**

All students aged 11–15 years enrolled in the selected school and present on the day of data collection were eligible to participate, subject to informed assent and parental consent. Teachers of the selected classes and parents of enrolled students were also invited to participate.

**Sample size and sampling**

As this was a pilot study, universal sampling was employed. All students aged 11–15 years enrolled in the selected school and present on the day of data collection were invited to participate. All teachers responsible for these classes were also included. Parents of enrolled students were invited to complete the questionnaire.

Tools

A brief structured questionnaire was developed with close-ended questions to assess the perceptions and practices related to HPV infection and vaccination. Given the exploratory objectives of the pilot, sociodemographic information was not collected. A single, common questionnaire was used for both parents and teachers to ensure uniformity and reduce respondent burden. Questionnaire items were informed by a review of existing KAP studies on HPV vaccination and were framed using simple, non-judgmental language to enhance comprehension and participation. Content validity was assessed through review by subject experts from Community Medicine and Biostatistics, who evaluated each item for relevance, clarity, and appropriateness for the target population. Face validity and feasibility were further examined through pilot testing among a small group of postgraduate students and subject experts, focusing on clarity, length, and ease of answering questions. Feedback from this process led to minor refinements, primarily aimed at shortening the questionnaire and improving wording. Internal consistency reliability of the final tool was assessed using Cronbach’s alpha, which demonstrated acceptable reliability for the overall questionnaire (Cronbach’s α ≥0.7), supporting its suitability for use in the pilot study.

**Data Collection** A structured questionnaire was developed in English, translated into Malayalam, and validated for content and face validity. Trained investigators administered the survey to students and teachers during school hours using paper-based forms. Parents were provided questionnaires through their wards. All completed forms were anonymized, coded, and entered into secure, password-protected electronic spreadsheets**.**

**Recruitment Period**

Data was collected between the 18^th^ and 19^th^ of June, 2025. Parental consent was taken on the first day, with the questionnaires and information sheet about the research sent home with the students for parental review. Student assent was taken on the second day before the questionnaires were filled in by them. Teacher consent was taken during the day of the study.

**Outcome Measures** The pilot study assessed the feasibility and clarity of items measuring knowledge of HPV and cervical cancer, awareness of the HPV vaccine, attitudes toward vaccination, willingness to receive or recommend the vaccine, perceived barriers, vaccination-related practices, and sources of information. These outcomes were used primarily to evaluate the performance of the questionnaire and identify areas requiring refinement for the follow up full-scale study.

**Data analysis**

Data were cleaned and analyzed using descriptive statistics to summarize sociodemographic characteristics and KAP outcomes. With no key demographic or behavioral variables available for multivariable modelling, no inferential or regression analyses were undertaken. The focus of analysis was to evaluate response patterns, identify missing or non-discriminating items, and assess the overall feasibility and suitability of the questionnaire for use in a larger study.

**Results**

Demographic Profile

The study was conducted at an Indian Certificate of School Education(ICSE) school at Pulinkunnu and included 75 students and 20 teachers. No parents participated, as no student returned the parent questionnaire, representing a deviation from the planned study design. Among the students, 16 (21.3%) were boys and 59 (78.7%) were girls. Among teachers, 2 (10%) were male and 18 (90%) were female. The majority of students were in the 10–12-year age group (45.3%), followed by 13–15 years (36%) and 16–17 years (18.7%). Most teachers were between 40–49 years (40%), with equal proportions in the 30–39 years and 50–59 years categories (30% each).

Table 1 : Age classification of the students and teachers

| Age Group | Frequency | Percentage |
| --- | --- | --- |
| 10-12 years | 34 | 45.3% |
| 13-15 years | 27 | 36% |
| 16-17 years | 14 | 18.7% |
|  | **75** |  |
| Teachers |  |  |
| 30-39 years | 6 | 30% |
| 40-49 years | 8 | 40% |
| 50-59 years | 6 | 30% |
|  | **20** |  |

Table 2 : Knowledge about HPV vaccines

| Question | Answer options | Students | Teachers |
| --- | --- | --- | --- |
| K1.HPV infection can cause cervical cancer | Yes | 38(50.7%) | 9(45%) |
|  | No | 37 (49.3%) | 11(55%) |
|  | Don’t know | 0 | 0 |
| K2.HPV is a sexually transmitted infection. | Yes | 38(50.7%) | 6 (30%) |
|  | No | 37 (49.3%) | 14 (70%) |
|  | Don’t know | 0 | 0 |
| K3. HPV affects only women. | Yes | 38 (50.7%) | 12 (60%) |
|  | No | 37 (49.3%) | 8 (40%) |
|  | Don’t know | 0 | 0 |
| K4. The HPV vaccine is most effective when given before sexual debut. | Yes | 38 (50.7 | 13 |
|  | No | 37 | 7 |
|  | Don’t know | 0 | 0 |
| K5. The HPV vaccine is available in India. | Yes | 38 | 7 |
|  | No | 37 | 13 |
|  | Don’t know | 0 | 0 |
| K6. The HPV vaccine protects against all types of HPV. | Yes | 35 | 9 |
|  | No | 40 | 11 |
|  | Don’t know | 0 | 0 |
| K7. Boys can also receive the HPV vaccine | Yes | 35 | 15 |
|  | No | 40 | 5 |
|  | Don’t know | 0 | 0 |
| K8. The HPV vaccine requires more than one dose. | Yes | 40 | 9 |
|  | No | 35 | 11 |
|  | Don’t know | 0 | 0 |
| K9. HPV infection can occur even without symptoms | Yes | 39 | 8 |
|  | No | 36 | 12 |
|  | Don’t know | 0 | 0 |
| K10. Pap smear is a screening test for cervical cancer. | Yes | 36 | 8 |
|  | No | 39 | 12 |
|  | Don’t know | 0 | 0 |
| K11. Do you know where to get the HPV vaccine? | Yes | 41 | 14 |
|  | No | 34 | 6 |

Table 3 : Attitude about HPV Vaccines

| Attitude Questions | Answer Options | Students | Teachers |
| --- | --- | --- | --- |
| A1. HPV vaccination should be included in the national immunization schedule. | Strongly Agree | 15 | 2 |
|  | Agree | 15 | 6 |
|  | Neutral | 19 | 4 |
|  | Disagree | 15 | 4 |
|  | Strongly Disagree | 11 | 4 |
| A2. I believe HPV vaccination is important for cancer prevention. | Strongly Agree | 11 | 2 |
|  | Agree | 12 | 4 |
|  | Neutral | 16 | 3 |
|  | Disagree | 15 | 5 |
|  | Strongly Disagree | 21 | 6 |
| A3. I would recommend the HPV vaccine to others. | Strongly Agree | 15 | 8 |
|  | Agree | 18 | 4 |
|  | Neutral | 10 | 3 |
|  | Disagree | 17 | 2 |
|  | Strongly Disagree | 14 | 3 |
| A4. I worry about the side effects of the HPV vaccine. | Strongly Agree | 19 | 3 |
|  | Agree | 14 | 8 |
|  | Neutral | 17 | 3 |
|  | Disagree | 10 | 2 |
|  | Strongly Disagree | 15 | 4 |
| A5. HPV vaccination promotes early sexual activity | Strongly Agree | 13 | 4 |
|  | Agree | 11 | 2 |
|  | Neutral | 20 | 5 |
|  | Disagree | 11 | 2 |
|  | Strongly Disagree | 20 | 7 |
| A6. I believe awareness campaigns are needed for HPV vaccination. | Strongly Agree | 14 | 1 |
|  | Agree | 13 | 3 |
|  | Neutral | 17 | 8 |
|  | Disagree | 13 | 5 |
|  | Strongly Disagree | 18 | 3 |
| A7. I trust the safety of the HPV vaccine. | Strongly Agree | 14 | 1 |
|  | Agree | 13 | 3 |
|  | Neutral | 17 | 8 |
|  | Disagree | 13 | 5 |
|  | Strongly Disagree | 18 | 3 |
| A8. If not vaccinated, are you willing to take it? | Yes | 33 | 8 |
|  | No | 42 | 12 |

Table 4 : Practice about HPV Vaccines

| Practice Questions | Answer Options | Students | Teachers |
| --- | --- | --- | --- |
| P1. Have you heard about the HPV vaccine before? | Yes | 45 | 10 |
|  | No | 30 | 10 |
| P2. Have you ever received the HPV vaccine ? | Yes | 39 | 10 |
|  | No | 36 | 10 |
| P3. Have you encouraged anyone to take the HPV vaccine? | Yes | 30 | 11 |
|  | No | 45 | 9 |

**1.** **Knowledge Interpretation ( Table 2)**

Low levels of knowledge of HPV and cervical cancer linkage

Only about half of students (50.7%) and teachers (45%) correctly identified that HPV causes cervical cancer. This indicates that foundational knowledge of HPV was inadequate in this school community.

Poor understanding of HPV transmission

Only 30% of teachers and half the students correctly identified HPV as a sexually transmitted infection. This was important because misunderstanding transmission pathways could weaken risk perception and minimize urgency for vaccination.

Significant misconceptions

Half the students and 60% of teachers incorrectly believed HPV affects only women. Almost half did not know the vaccine was available in India. Many believed HPV infection always presented with symptoms. These misconceptions implied that health education on HPV in schools had not been effectively integrated, and teachers themselves required orientation before offering student guidance.

Limited knowledge of screening methods

Only 36 students and 8 teachers knew Pap smear was used for cervical cancer screening. This suggested major gaps in understanding of the prevention continuum (screening + vaccination) for HPV cervical cancer.

Better awareness about vaccination eligibility for boys

Students and teachers showed comparatively better awareness that boys can receive HPV vaccines. This was encouraging and aligning with current recommendations.

Overall, knowledge levels were low-to-moderate, with several misunderstandings that could impede vaccine uptake.

**2.** **Attitude Interpretation( Table 3)**

“Neutral” and “hesitant” attitudes dominate

Most respondents did not strongly support HPV vaccination; neutral responses were common across nearly all attitude items. This indicated uncertainty rather than rejection.

Concerns about side effects were high

Around one-third strongly agreed/agreed that they worried about vaccine side effects ( Question A4).

Belief that HPV vaccination may promote early sexual activity

Approximately 32% of students and teachers agreed or strongly agreed with this misconception. This reflected socio-cultural norms and moral concerns, which have historically influenced vaccine hesitancy.

Mixed trust in vaccine safety

Many respondents remained neutral or disagreed that HPV vaccines were safe. This suggested the need for targeted communication addressing safety, effectiveness, and global evidence of long-term benefit.

Willingness to be vaccinated

Despite low knowledge levels, 44% of students and 40% of teachers expressed willingness to take the vaccine if not already vaccinated. This indicated that improving awareness could translate into greater uptake.

From the responses to the “Attitude” questions, there seems to be a “hesitant to vaccine-uptake” population, where attitude shifts through education are required**.**

**3.** **Practice Interpretation**

Moderate awareness but low actual vaccination

While 45 students and 10 teachers had heard of HPV vaccines, only 39 students and 10 teachers had received it.
 This mismatch suggested barriers to access vaccines, low parental support, safety concerns about the HPV vaccine, and limited communication from schools or healthcare providers due to ‘vaccine shyness’, or sociocultural factors that prevent them from talking about the vaccine.

Low rates of recommending vaccination to others

Only 30 students and 11 teachers reported encouraging someone else to take the vaccine.
 This aligned with their low confidence and incomplete knowledge, possibly reflecting the unsurety of individuals about the HPV vaccine safety and necessity.

Absence of parental participation

No parents participated in the survey . This highlighted difficulty involving parents—who are the key decision-makers for adolescent vaccination.

Overall, HPV vaccine practices are poor, reflecting the knowledge deficits and hesitant attitudes and low practice parameters.

Demographic Interpretation

Majority of students were girls, which might have influenced responses as cervical cancer is often perceived as a “female-only” issue. Teachers were predominantly female and in mid-career age groups; traditionally such groups were expected to support adolescent health programs, yet knowledge levels among this cohort were low. This suggested the need for structured teacher training before implementing school-based HPV initiatives.

Insights from the pilot study to guide a scale-up study

Though levels of knowledge, attitudes, and self-reported practices about the HPV vaccine could be described, the authors realized the study was unable to identify meaningful predictors of good knowledge, positive attitudes, or favorable practices related to HPV vaccination, particularly those operating at the parental, household, school, and socioeconomic levels as these variables were either absent or inadequately captured.

From a feasibility perspective, the pilot demonstrated that though KAP studies are good to capture the reasons behind health decisions at a point in time, not capturing contextual and decision-making variables limits interpretability. Parent and teacher practices around HPV vaccination are likely influenced by complex social, cultural, and informational factors that are not easily elicited through closed-ended questions. Interview-based or mixed-methods approaches may be necessary to understand how adults interpret vaccine information, perceive risk, and make decisions for adolescents. These insights underscored the need to reframe the questionnaire and to incorporate qualitative methods in the main study.

**Discussion**

This school-based study provides critical insights into the knowledge, attitudes, and practices related to HPV vaccination among students and teachers. The findings reveal substantial gaps in awareness, widespread misconceptions, and low vaccination uptake despite moderate exposure to HPV-related information. When examined alongside the national and global evidence base, the results highlight systemic information gaps, sociocultural influences, and structural barriers that continue to limit HPV vaccine acceptability and uptake in India.

Knowledge Levels Compared with Other Studies

In this study, only about half of students and teachers recognized that HPV causes cervical cancer. These findings align with several Indian studies demonstrating poor foundational knowledge about HPV. Backer et al. (2024) reported that although 85.6% of Kerala college students knew HPV was linked to cervical cancer, only 48.6% had heard of the vaccine.(20) Similar patterns were seen in North India, where Basu et al. found that adolescents were largely unaware of HPV transmission and its association with cervical cancer. (21) Internationally, limited awareness is also well-documented in Low Middle-Income Countries( LMIC), particularly where sexual health education is restricted. (30, 31)

A major misconception observed was the belief that HPV affects only women. This misconception has been highlighted globally. Studies from Europe and the United States show that boys often underestimate their susceptibility to HPV-related diseases, including penile, oropharyngeal, and anal cancers. (32, 33) This gendered perception reduces motivation for male vaccination, despite WHO and CDC recommendations for universal vaccination of both sexes. (34-36)

Similarly, inadequate awareness that the HPV vaccine is available in India reflects a lack of systematic public communication. Many studies have found that even medical students were uncertain about vaccination availability and access points. (37, 38) In contrast, Sikkim’s school-based Programme achieved high awareness due to intensive pre-implementation education. (12) The contrast indicates that Kerala has not yet conducted similar structured outreach.

Overall, the knowledge gap in our study is consistent with national trends and highlights the absence of integrated HPV education in schools, even in high-literacy states like Kerala.

Misconceptions and Underlying Influences

Fear of HPV vaccine side effects was prominent in our study, echoing findings from several Indian and global studies. Seo et al. reported through their scoping review that concerns about safety and adverse events are the leading reason for HPV vaccine refusal among parents.(39) Asmelash et al. similarly identified safety concerns as a major determinant of vaccine hesitancy across West and East African countries. (40)Such concerns are often fueled by misinformation, lack of transparent communication, and limited exposure to scientific evidence.

Another recurring misconception was the belief that HPV vaccination may promote early sexual activity. This has been repeatedly shown to be untrue in scientific literature, yet remains a widespread cultural fear. Studies in Türkiye, Africa, and South Korea report that parents worry vaccination may be interpreted as permission for sexual activity.(41-43) Studies from the US, however, show no association between HPV vaccination and sexual Behaviour . (44)The persistence of this misconception highlights cultural discomfort with sexuality education, particularly in conservative settings.

Misconceptions about the number of doses required and uncertainty about asymptomatic HPV infections further indicate the absence of structured, accurate health communication in schools. Similar misunderstandings have been reported in East Asian and African countries, where lack of clarity about vaccine schedules hinders adherence. (42, 45-47)

Attitudes Toward HPV Vaccination in Context

The predominance of “neutral” or “uncertain” attitudes in our study indicates “ambivalent” hesitancy rather than outright refusal. This is widely observed in vaccine acceptance research. Lelliott et al. describe ambivalence as a form of “information hesitancy,” where individuals are unsure due to incomplete knowledge rather than opposition.(48) Backer et al. and Muthukrishnan et al. also report that unclear, inconsistent, or conflicting information contributes to passive resistance to vaccination.(20, 49)

Safety concerns were common in our sample, consistent with a recent study by Waheed et al., and an early study by Holman, which identified perceived risk of adverse events as the most influential barrier to HPV vaccination globally. (50, 51) Conversely, countries with long-standing HPV vaccination programmes—such as Australia and the United Kingdom—report significantly lower safety-related hesitancy because of strong public trust and robust adverse event monitoring. (52, 53)

Importantly, willingness to be vaccinated was relatively high once individuals were informed, indicating that hesitancy is modifiable. Aslemash et al. demonstrated that even brief educational interventions significantly increase willingness to vaccinate. (40)Similar improvements have been documented in US and European settings. (54) Thus, the results of our study suggest that structured school-based awareness programmes could rapidly improve acceptance in Pulinkunnu.

Practices and the Knowledge–Behaviour Gap

Despite moderate awareness, actual vaccination uptake remained low among both students and teachers. This gap between intention and practice is well documented in India. Basu et al. found that even when adolescents expressed interest, parental concerns, cost, and lack of access remained barriers. (21) Das et al. reported low uptake even among medical students, demonstrating that knowledge in some settings does not directly translate to action.(18)

The absence of parental participation in our study further highlights a major barrier. Numerous studies have shown that parental consent and beliefs are the strongest predictors of adolescent HPV vaccination. In South India, for example, parental attitudes accounted for more than 50% of vaccination decisions. (27, 55)The complete absence of parental responses here underscores the urgent need to involve families in HPV awareness initiatives.

When compared with successful models, the gap becomes clearer. Sikkim’s Programme and the Happy Noolpuzha campaign in Wayanad achieved high coverage through community mobilization, strong local leadership, and parent-centered communication. (12, 19) Our findings suggest that without such community engagement, school-based programmes alone may be insufficient.

**Proposed Changes to existing questionnaires in preparation for future research**

**a.**	**Add variables that can act as predictors**

Sociodemographic predictors like parental level of education , occupation and socioeconomic status, family structure and decision-maker for child vaccination in families, and prior exposure to health campaigns or school health programs are all important predictors which must be studied. Specific health system and information exposure questions need be added like source of HPV vaccine information, level of trust in healthcare providers and government programs, previous experience with childhood or adolescent vaccinations, or awareness of government HPV vaccination policies. Cultural and belief-based factors are another dimension to be explored like perceived stigma around sexually transmitted infections, comfort discussing sexual health topics, perceived moral or cultural concerns related to HPV vaccination, gender norms influencing adolescent health decisions.

b. **Modify data-collection mode**

The pilot indicated that self-administered online questionnaires may not be optimal, particularly for parents, as literacy and digital familiarity may affect response quality. The following methodological changes are being proposed : interviewer-administered, short, semi-structured questionnaires for parents and teachers where possible to explore reasoning behind practices and capture unanticipated concerns or motivations. These would enhance data completeness, depth, and validity.

Public Health Implications

The findings of this study highlight the requirement for three strategic actions. School based health education with structured sessions for teachers, parents, and students addressing safety, immunization schedules, benefits, costs, adverse events, and availability of the HPV are required. Parental involvement with regular meetings, and open house sessions with educational brochures for clarifying doubts are recommended. The places where HPV vaccines are available, the methods to procure the vaccine etc. should be provided to ensure accessibility and clarity for referral. These strategies align with WHO’s elimination framework and the national plan for HPV vaccination under the Universal Immunization Program( UIP).

Strengths of the pilot study

A key strength of this pilot study lies in its feasibility-driven design. Conducted as a preliminary investigation, the study was essential for refining the student, teacher, and parent questionnaires, particularly to identify and restructure items capturing key predictor variables for HPV vaccine knowledge, attitudes, and acceptance. The use of a validated questionnaire, combined with insights gained from pilot testing, enhanced content clarity, contextual relevance, and construct coverage for the main study.

The pilot study conducted among 75 students and 20 teachers in a socio-demographically comparable setting—the neighboring district of Alappuzha( district neighboring to Pathanamthitta district where the full study is planned)—allowed assessment of recruitment processes, participation rates, and data-collection logistics without contaminating the main study population. The pilot also provided realistic estimates of time, personnel, and material resources required for large-scale implementation, thereby strengthening the methodological preparedness of the primary study.

Limitations include the absence of parent responses during the pilot phase, the single-school setting, and reliance on self-reported practices, which may be subject to reporting bias. Online or self-filled formats (e.g., Google Forms) were associated with incomplete responses, superficial answers, and difficulty probing reasoning behind decisions.

However, consistent with the purpose of pilot and feasibility studies, the findings are not intended to be generalizable but to inform study design and implementation. The study nevertheless provides valuable baseline insights from a rural context relevant to HPV vaccine introduction.

Conclusion

The study demonstrates major knowledge gaps, mixed attitudes, and low HPV vaccination uptake among students and teachers, though willingness improves when informed. Strengthening school-based awareness, involving parents, and improving access to vaccination services will be critical for successful Programme implementation. With national HPV rollout underway, these findings can guide local action for cervical cancer prevention.

Key findings

| Domain | Observations | Implication |
| --- | --- | --- |
| Knowledge | ~50% knew HPV causes cervical cancer | Large gaps despite high literacy |
| Misconceptions | HPV affects only women; vaccine not available | Targeted correction needed |
| Attitudes | Neutral or uncertain responses dominant | Hesitancy rather than rejection |
| Safety Concerns | Common among both groups | Need for counselling |
| Uptake | Low vaccination despite willingness | Access and family involvement critical |

References

1.	Trottier H, Franco EL. The epidemiology of genital human papillomavirus infection. Vaccine. 2006;24 Suppl 1:S1-15.

2.	IARC. IARC Working Group on the Evaluation of Carcinogenic Risks to Humans. Human Papillomaviruses. Lyon (FR): International Agency for Research on Cancer; 2007; 2007.

3.	Vanden Broeck D e. Human Papillomavirus and Related Diseases - From Bench to Bedside - Research aspects 2012 25 January 2012.

4.	Bouvard V, Baan R, Straif K, Grosse Y, Secretan B, El Ghissassi F, et al. A review of human carcinogens--Part B: biological agents. Lancet Oncol. 2009;10(4):321-2.

5.	Senapati R, Nayak B, Kar SK, Dwibedi B. HPV Genotypes distribution in Indian women with and without cervical carcinoma: Implication for HPV vaccination program in Odisha, Eastern India. BMC Infect Dis. 2017;17(1):30.

6.	de Martel C, Plummer M, Vignat J, Franceschi S. Worldwide burden of cancer attributable to HPV by site, country and HPV type. Int J Cancer. 2017;141(4):664-70.

7.	Organization WH. Cervical cancer [Online]. Geneva: WHO; 2025 [updated December 2, 2025. Available from: <https://www.who.int/news-room/fact-sheets/detail/cervical-cancer>.

8.	Mailhot Vega RB, Balogun OD, Ishaq OF, Bray F, Ginsburg O, Formenti SC. Estimating child mortality associated with maternal mortality from breast and cervical cancer. Cancer. 2019;125(1):109-17.

9.	BGI. BGI Genomics 2023 Global State of Cervical Cancer Awareness Report. 2023.

10.	Dwivedi P, Lahariya AU, Pandey S, Sreedevi A, Lohiya A, Lahariya C. Cervical Cancer and Human Papilloma Virus (HPV) Vaccines: A primer for General Physicians. Preventive Medicine: Research & Reviews. 2024;1(3):157-61.

11.	WHO. Global strategy to accelerate the elimination of cervical cancer as a public health problem. 2020 [Available from: <https://www.who.int/publications/i/item/9789240014107>.

12.	Ahmed D, VanderEnde K, Harvey P, Bhatnagar P, Kaur N, Roy S, et al. Human papillomavirus (HPV) vaccine introduction in Sikkim state: Best practices from the first statewide multiple-age cohort HPV vaccine introduction in India-2018-2019. Vaccine. 2022;40 Suppl 1(Suppl 1):A17-A25.

13.	Public Health Faculty Launch HPV Vaccination Project in Southern India [press release]. Arizona: Mel and Enid Zuckerman College of Public Health, January 22, 2024 2024.

14.	NDTV. Made In India HPV Vaccine May Be Considered For Immunization: Centre. New Delhi: Syndicated Feed; 2023.

15.	OManorama. Vaccination against cervical cancer; pilot project to be launched in Wayanad and Alappuzha. OManorama [Internet]. 2024. Available from: <https://www.onmanorama.com/lifestyle/health/2024/02/09/kerala-cervical-cancer-alappuzha-wayanad-hpv-vaccine.html>.

16.	India ETo. HPV vaccine not included in Universal Immunization Programme, Nadda tells LS 2024. Available from: <https://health.economictimes.indiatimes.com/news/policy/hpv-vaccine-not-included-in-universal-immunization-programme-nadda-tells-ls/116045203>.

17.	Online K. Cancer vaccination for plus two students; vaccine worth Rs vaccine worth Rs 2000 for free 2024. Available from: <https://keralakaumudi.com/en/news/news.php?id=1245387&u=cancer-vaccination-for-plus-two-students-vaccine-worth-rs.2000-for-free-1245387>.

18.	Das E. N, & Francis, P. T. . HPV vaccine knowledge and coverage among female students in a medical college, Kerala. . International Journal Of Community Medicine And Public Health. 2018;5(12):5133–8. .

19.	Hindu T. Happy Noolpuzha Initiative : Combating cervical cancer through HPV vaccination2023. Available from: <https://www.thehindu.com/news/cities/Kochi/vaccine-programme-to-prevent-cervical-cancer-on-the-anvil-cm-vijayan/article67371697.ece>.

20.	Backer A, Sudha, A. V., Suresh, A., Subhana, A., & Ahammed, D. . Awareness and attitude towards human papillomavirus vaccination for cervical cancer prevention among college students in South Kerala. . International Journal Of Community Medicine And Public Health, (2024). 11((11)): 4270–4. .

21.	Basu P, Malvi SG, Joshi S, Bhatla N, Muwonge R, Lucas E, et al. Vaccine efficacy against persistent human papillomavirus (HPV) 16/18 infection at 10 years after one, two, and three doses of quadrivalent HPV vaccine in girls in India: a multicenter, prospective, cohort study. Lancet Oncol. 2021;22(11):1518-29.

22.	Dempsey AF, O'Leary ST. Human Papillomavirus Vaccination: Narrative Review of Studies on How Providers' Vaccine Communication Affects Attitudes and Uptake. Acad Pediatr. 2018;18(2S):S23-S7.

23.	Dubé E, Gagnon D, Pelletier C, Comeau JL, Steenbeek A, MacDonald N, et al. Enhancing HPV vaccine uptake in girls and boys - A qualitative analysis of Canadian school-based vaccination programs. Vaccine. 2024;42(26):126425.

24.	Aggarwal S, Agarwal P, Gupta N. A comprehensive narrative review of challenges and facilitators in the implementation of various HPV vaccination program worldwide. Cancer Med. 2024;13(3):e6862.

25.	Apurva U T MPT, Malangori A P, Poonam V S, Pradip S B. Effect of Health Education on the Knowledge and Attitude Regarding the Human Papillomavirus Vaccine among Adolescent School Girls of a City in Western Maharashtra. . Indian Journal of Community Health [Internet]. 2024;36(4).

26.	Nalawade K, Bajaj, P., Bajaj, U., & Meher, A. . Impact of a health education session on the knowledge of Human Papillomavirus Vaccination (HPV) among the school teachers. International Journal of Health Sciences. 2024;8((S1)):1188–200. .

27.	Degarege A, Krupp K, Fennie K, Li T, Stephens DP, Marlow LAV, et al. Urban-Rural Inequities in the Parental Attitudes and Beliefs Towards Human Papillomavirus Infection, Cervical Cancer, and Human Papillomavirus Vaccine in Mysore, India. J Pediatr Adolesc Gynecol. 2018;31(5):494-502.

28.	Pearson N, Naylor PJ, Ashe MC, Fernandez M, Yoong SL, Wolfenden L. Guidance for conducting feasibility and pilot studies for implementation trials. Pilot Feasibility Study. 2020;6(1):167.

29.	Eldridge SM, Chan CL, Campbell MJ, Bond CM, Hopewell S, Thabane L, et al. CONSORT 2010 statement: extension to randomized pilot and feasibility trials. BMJ. 2016;355:i5239.

30.	Reiter PL, Gilkey MB, Brewer NT. HPV vaccination among adolescent males: results from the National Immunization Survey-Teen. Vaccine. 2013;31(26):2816-21.

31.	Bhoopathi V, Bhagavatula P, Singh M. Health literacy and its association with human papilloma virus vaccination among adults: Findings from the behavioral risk factor surveillance system. Hum Vaccin Immunother. 2022;18(6):2135930.

32.	Lopez N, Salamanca de la Cueva I, Verges E, Suarez Vicent E, Sanchez A, Lopez AB, et al. Factors influencing HPV knowledge and vaccine acceptability in parents of adolescent children: results from a survey-based study (KAPPAS study). Hum Vaccin Immunother. 2022;18(1):2024065.

33.	Lacombe-Duncan A, Newman PA, Baiden P. Human papillomavirus vaccine acceptability and decision-making among adolescent boys and parents: A meta-ethnography of qualitative studies. Vaccine. 2018;36(19): 2545-58.

34.	WHO. WHO. Human papillomavirus vaccines: WHO position paper Geneva: WHO; 2017 [19:[2017;92(19):241–68].

35.	CDC. HPV Vaccination Recommendations Atlanta: CDC; [Available from: <https://www.cdc.gov/vaccines/vpd/hpv/hcp/recommendations.html>.

36.	Updates W. WHO updates recommendations on ?HPV vaccination schedule: WHO; 2022 [Available from: <https://www.who.int/news/item/20-12-2022-WHO-updates-recommendations-on-HPV-vaccination-schedule>.

37.	Bhatia N, Sri Harika B, S Reddy R. Awareness and attitude towards HPV vaccine among medical students. Indian Journal of Obstetrics and Gynecology Research. 2025;12(4):686-92.

38.	M R, Sadiqunissa S, Ahmed M. Awareness and knowledge of human papilloma virus (HPV) vaccine in prevention of cervical cancer among medical students. International Journal of Reproduction, Contraception, Obstetrics and Gynecology. 2018;7(12).

39.	Seo J, Choi Y, Yang H, Seo H-J. Understanding facilitators and barriers of HPV vaccination amongst adolescent boys and their parents in high-income countries through the social-ecological model: a scoping review. Health Education Research. 2025;40(6).

40.	Asmelash D, Zewdia WF, Abebe GF, Girma D, Mohammed AH, Asres A. Prevalence and predictors of parental willingness to vaccinate daughters against human papillomavirus in Sub-Saharan Africa: a systematic review and meta-analysis. Front Public Health. 2025;13:1486262.

41.	Singh G, Dash NR, Shaju A, Chakkalakkunnan SS. Perceptions and sentiments associated with HPV vaccine uptake among Indian Reddit users: a qualitative social media analysis. BMC Public Health. 2025;25(1):4037.

42.	Alene T, Atnafu A, Mekonnen ZA, Minyihun A. Acceptance of Human Papillomavirus Vaccination and Associated Factors Among Parents of Daughters in Gondar Town, Northwest Ethiopia. Cancer Manag Res. 2020;12:8519-26.

43.	Choi J, Markham C, Tami-Maury I, Kim S, Cuccaro P. Maternal perceptions of vaccinating boys against human papillomavirus (HPV) in Seoul, South Korea: A descriptive exploratory qualitative study. PLoS One. 2023;18(3):e0282811.

44.	Leidner AJ, Chesson HW, Talih M. HPV vaccine status and sexual behavior among young sexually-active women in the US: evidence from the National Health and Nutrition Examination Survey, 2007-2014. Health Econ Policy Law. 2020;15(4):477-95.

45.	Uzunlar Ö, Özyer Ş, Başer E, Toğrul C, Karaca M, Güngör T. A survey on human papillomavirus awareness and acceptance of vaccination among nursing students in a tertiary hospital in Ankara, Turkey. Vaccine. 2013;31(17):2191-5.

46.	Endarti D, Satibi S, Kristina SA, Farida MA, Rahmawanti Y, Andriani T. Knowledge, Perception, and Acceptance of HPV Vaccination and Screening for Cervical Cancer among Women in Yogyakarta Province, Indonesia. Asian Pac J Cancer Prev. 2018;19(4):1105-11.

47.	You D, Han L, Li L, Hu J, Zimet GD, Alias H, et al. Human Papillomavirus (HPV) Vaccine Uptake and the Willingness to Receive the HPV Vaccination among Female College Students in China: A Multicenter Study. Vaccines (Basel). 2020;8(1).

48.	Lelliott M, Sahker E, Poudyal H. A Review of Parental Vaccine Hesitancy for Human Papillomavirus in Japan. J Clin Med. 2023;12(5).

49.	Muthukrishnan M, Loux T, Shacham E, Tiro JA, Arnold LD. Barriers to human papillomavirus (HPV) vaccination among young adults, aged 18-35. Prev Med Rep. 2022;29:101942.

50.	Waheed DE, Olivier CW, Riethmuller D, Franco EL, Prétet JL, Baay M, et al. Prevention and control of HPV and HPV-related cancers in France: the evolving landscape and the way forward - a meeting report. BMC Proc. 2023;17(Suppl 11):18.

51.	Holman DM, Benard V, Roland KB, Watson M, Liddon N, Stokley S. Barriers to human papillomavirus vaccination among US adolescents: a systematic review of the literature. JAMA Pediatr. 2014;168(1):76-82.

52.	Phillips A, Hickie M, Totterdell J, Brotherton J, Dey A, Hill R, et al. Adverse events following HPV vaccination: 11 years of surveillance in Australia. Vaccine. 2020;38(38):6038-46.

53.	Nurses. HPV-school-nurse_factsheet-cervical-cancer.pdf>. In: UKHSA, editor. 2024.

54.	Gray A, Fisher CB. Factors associated with HPV vaccine acceptability and hesitancy among Black mothers with young daughters in the United States. Front Public Health. 2023;11:1124206.

55.	Punita Kumari SK, Bhupendra Narayan, Rupam Sinha. Attitudes and Perceptions of HPV Vaccine in North Indian Adolescent Girls and Their Parents. International Journal of Life Sciences, Biotechnology and Pharma Research. 2024;13(6).
