# Supplementary material for: Perceptions and practices among students, parents, and teachers of the Human Papillomavirus (HPV) Vaccination: A pilot feasibility study from Rural Kerala
Source: PLoS One. 2026 Aug 10;21(8):e0340559. doi: 10.1371/journal.pone.0340559 (PMC13456282; doi:10.1371/journal.pone.0340559)
Supplement: Supplementary file 8 [file pone.0340559.s010.docx]

Dear Editors,


Thank you for the detailed review of our manuscript and the time spent on looking through the manuscript multiple times to ensure it is an article that is as error-free as possible. Thank you for handholding us through the entire process. We have tried to make all the changes suggested to our manuscript and hope this meets the requirements of the Editors and the Journal.

We have included the following items with the revised manuscript:

- A letter that responds to each point raised by the academic editor and reviewer(s) has been included and labelled 'Response to Reviewers'.
- A marked-up copy of your manuscript that highlights changes made to the original version is uploaded as a separate file labelled 'Revised Manuscript with Track Changes'.
- An unmarked version of your revised paper without tracked changes is uploaded this as a separate file labelled 'Manuscript'.

1. Decision: Minor Revision

   Executive Summary
   The authors have conducted a thorough and responsive revision addressing the major methodological, ethical, and reporting concerns raised in the prior review. The manuscript is substantially improved: the abstract now correctly frames the pilot’s feasibility objectives and explicitly reports zero parent participation; the questionnaire and anonymized dataset are provided as supplementary files; Cronbach’s alpha values are reported with subscale breakdowns; interpretive material has been moved from Results to Discussion; and a dedicated “Lessons Learned for Future Research” section has been added. The authors have also moderated overgeneralizations and strengthened limitations regarding gender imbalance, single-school setting, and social desirability bias. However, despite these commendable improvements, several residual issues require attention before the manuscript can be accepted for publication. These are minor but necessary corrections to ensure clarity, consistency, and compliance with journal standards.

Response: We thank the reviewer for this helpful observation and for their positive assessment of the revisions made to the manuscript. We agree that this issue required clarification. Accordingly, we have revised the manuscript and relevant changes have been incorporated in the revised manuscript.

Minor Revisions
1. Inconsistent numerical data in Tables (Critical for reproducibility)
• Table 2 (Knowledge):

a. For K4 (HPV vaccine most effective before sexual debut), student responses show “Yes 38 (50.7)” and “No 37” – but 38+37 = 75, yet the percentage for “Yes” is correctly 50.7%. Please verify and align all such pairs to avoid any perception of rounding errors.
b. Table 2, K11: “Do you know where to get the HPV vaccine?” – Student “Yes” = 41 (54.7%?) but denominator is 75. Current text shows “Yes 41, No 34” (sum 75). Please recalculate percentage and ensure consistency with the 95% CI added in the revised manuscript (which currently shows different values in the track-changes version vs. clean version).

Response: We thank the reviewer for this careful review of the numerical data and for highlighting these inconsistencies.

a. K4 (HPV vaccine most effective before sexual debut)

We rechecked the original dataset and verified that the correct student responses for K4 are: Yes = 35 (46.7%) and No = 40 (53.3%), which sum to the total sample of 75 students. The values referred to by the reviewer (Yes = 38 and No = 37) were present in an earlier tracked-changes version and do not reflect the final dataset. We have carefully reviewed the table and ensured that the frequencies, percentages, and corresponding 95% confidence intervals are consistent throughout the revised manuscript and supplementary materials.

b. K11 (Do you know where to get the HPV vaccine?)

We thank the reviewer for identifying this issue. Upon verification against the original dataset, the correct student responses are: Yes = 40 (53.3%) [41.4–64.9] and No = 35 (46.7%) [35.1–58.6], based on a denominator of 75 students. The discrepancy noted by the reviewer arose from differences between an earlier tracked-changes version and the final clean version of the manuscript. We have rechecked all frequencies, percentages, and 95% confidence intervals in Table 2 and confirmed that the values reported in the revised manuscript are correct and internally consistent.

In addition, we conducted a comprehensive review of all tables, percentages, confidence intervals, and supplementary files to ensure complete consistency and reproducibility across the manuscript.

•Table 4, P2: “Have you ever received the HPV vaccine?” For students, “Yes 39 (52.0%), No 36 (48.0%)” – correct. But the table in the clean manuscript (page 54) erroneously repeats “39 (52.0%)” twice under the teacher column. Teacher responses should be “Yes 10 (50.0%), No 10 (50.0%)” as shown elsewhere. Please correct.

Response:

We thank the reviewer for identifying this error and are very sorry this was not picked up during our review of the manuscript.

Upon verification against the original dataset, we confirmed that a typographical error occurred in Table 4 (P2: “Have you ever received the HPV vaccine?”). The teacher column incorrectly repeated the student value “39 (52.0%) [40.2–63.6]”. The correct teacher responses are:

Yes = 10 (50.0%) [27.2–72.8]

No = 10 (50.0%) [27.2–72.8]

The table has been corrected accordingly in the revised manuscript. We have also rechecked all frequencies, percentages, and confidence intervals reported in Table 4 against the original dataset to ensure accuracy and internal consistency.

2. Missing in-text citation for Supplementary Table S4
• In the clean manuscript (page 37), you state that the summary table was moved to Supporting Information as S4 Table. However, there is no in-text citation to S4 Table anywhere in the revised manuscript. Please add a citation (e.g., in the Discussion or Conclusions) to direct readers to this summary.

Response: Thank you- we have added a statement towards the end of the first paragraph of the Discussion to reflect this. “A summary of the study findings is presented in Supporting Information Table S4.”

3. Redundant text in the “Practices Related to HPV Vaccination” section
• Page 54 (clean manuscript), Table 4 is immediately followed by a second, incomplete Table 4 fragment. Please delete the duplicate fragment beginning with “Table 4. HPV vaccine awareness…” that appears after the correct table.

Response: We are very sorry this happened and we have removed the half-table that followed the Table 4 in the clean document.

4. Typographical and grammatical corrections
• Page 22 (abstract): Remove the extra comma: “major gaps in HPV-related knowledge, attitudes, and practices, among stakeholders” → delete comma after “practices.”

Comments: The corrected sentence has been highlighted in the manuscript. Thank you.
• Page 31 (Discussion): “The study suggests that the understanding … remain incomplete” → “remains incomplete” (subject-verb agreement).

Response: Thank you, the correction has been made.

• Page 32: “Aslemash et al.” should be “Asmelash et al.” (consistent with reference 40).

Response: Thank you, the correction has been made.

• Page 35 (Lessons Learned, section a): The list begins with “1. Add socioeconomic…” but subsequent sub-sections use “b., c., d.” – change “1.” to “a.” for consistency.

Response: Thank you, the correction has been made.

• Page 60 (reference 18): Remove the extra space and period: “5133–8.” → “5133–8.”

Response: Thank you, the correction has been made.

5. Data Availability Statement
• The statement says data are provided as Supporting Information. Please confirm that the anonymized dataset file has been uploaded with the revision and is cited as a supplementary file (e.g., S5 File or similar). Currently, I see references to S1-S4 Tables but no reference to a separate data file in the manuscript text.

Response: The anonymized data file is provided as an Excel sheet and though labelled as Supplementary file is attached in Excel Format as a separate file. (**S3 File** – Fully anonymized dataset)

6. Minor formatting inconsistency
• In the clean manuscript, some section headings (e.g., “Materials and methods” on page 25) use sentence case, while others (e.g., “Results” on page 27) use title case. Please standardize to PLOS ONE style (title case for main sections).

Response: Thank you, the correction has been made.

Recommendation
The manuscript now meets the essential standards for a pilot feasibility study report in PLOS ONE. The topic is timely and relevant, the revisions have been responsive and good-faith, and the lessons learned will genuinely inform future research. Once the above minor corrections are completed, the manuscript will be acceptable for publication.

Respected sir, thank you for your very gracious and thorough examination of our manuscript and the time taken to re-review it before final approval. We appreciate the time and effort you have put in to help make this manuscript even better.

Warm Regards,
Anjum

Date: June 12, 2026
