# Supplementary material for: Perceptions and practices among students, parents, and teachers of the Human Papillomavirus (HPV) Vaccination: A pilot feasibility study from Rural Kerala
Source: PLoS One. 2026 Aug 10;21(8):e0340559. doi: 10.1371/journal.pone.0340559 (PMC13456282; doi:10.1371/journal.pone.0340559)
Supplement: Peer review history

# Editor report: Decision Letter 0

**DOI:** 10.1371/journal.pone.0340559.r001

**Authors:** Morufu Raimi

13 Jan 2026

PONE-D-25-67419

Perceptions and Practices Among Students, Parents, and Teachers of the Human Papilloma Virus( HPV) vaccine : A Pilot Feasibility  Study from Rural Kerala

PLOS One

Dear Dr. John,

Thank you for submitting your manuscript to PLOS ONE. After careful consideration, we feel that it has merit but does not fully meet PLOS ONE’s publication criteria as it currently stands. Therefore, we invite you to submit a revised version of the manuscript that addresses the points raised during the review process.

Please submit your revised manuscript by Feb 27 2026 11:59PM. If you will need more time than this to complete your revisions, please reply to this message or contact the journal office at plosone@plos.org. When you're ready to submit your revision, log on to https://www.editorialmanager.com/pone/ and select the 'Submissions Needing Revision' folder to locate your manuscript file.

Please include the following items when submitting your revised manuscript:

- A letter that responds to each point raised by the academic editor and reviewer(s). You should upload this letter as a separate file labeled 'Response to Reviewers'.
- A marked-up copy of your manuscript that highlights changes made to the original version. You should upload this as a separate file labeled 'Revised Manuscript with Track Changes'.
- An unmarked version of your revised paper without tracked changes. You should upload this as a separate file labeled 'Manuscript'.

If you would like to make changes to your financial disclosure, please include your updated statement in your cover letter. Guidelines for resubmitting your figure files are available below the reviewer comments at the end of this letter.

If applicable, we recommend that you deposit your laboratory protocols in protocols.io to enhance the reproducibility of your results. Protocols.io assigns your protocol its own identifier (DOI) so that it can be cited independently in the future. For instructions see: https://journals.plos.org/plosone/s/submission-guidelines#loc-laboratory-protocols. Additionally, PLOS ONE offers an option for publishing peer-reviewed Lab Protocol articles, which describe protocols hosted on protocols.io. Read more information on sharing protocols at https://plos.org/protocols?utm_medium=editorial-email&utm_source=authorletters&utm_campaign=protocols.

We look forward to receiving your revised manuscript.

Kind regards,

Morufu Olalekan Raimi

Academic Editor

PLOS One

Journal Requirements:

When submitting your revision, we need you to address these additional requirements.

1. Please ensure that your manuscript meets PLOS ONE's style requirements, including those for file naming. The PLOS ONE style templates can be found at https://journals.plos.org/plosone/s/file?id=wjVg/PLOSOne_formatting_sample_main_body.pdf and https://journals.plos.org/plosone/s/file?id=ba62/PLOSOne_formatting_sample_title_authors_affiliations.pdf

2. Please include your full ethics statement in the ‘Methods’ section of your manuscript file. In your statement, please include the full name of the IRB or ethics committee who approved or waived your study, as well as whether or not you obtained informed written or verbal consent. If consent was waived for your study, please include this information in your statement as well.

3. In the online submission form, you indicated that the data cannot be shared publicly because of privacy reasons but they are available with the authors and will be provided anonymously, upon reasonable request.

All PLOS journals now require all data underlying the findings described in their manuscript to be freely available to other researchers, either 1. In a public repository, 2. Within the manuscript itself, or 3. Uploaded as supplementary information.

This policy applies to all data except where public deposition would breach compliance with the protocol approved by your research ethics board. If your data cannot be made publicly available for ethical or legal reasons (e.g., public availability would compromise patient privacy), please explain your reasons on resubmission and your exemption request will be escalated for approval.

4. When completing the data availability statement of the submission form, you indicated that you will make your data available on acceptance. We strongly recommend all authors decide on a data sharing plan before acceptance, as the process can be lengthy and hold up publication timelines. Please note that, though access restrictions are acceptable now, your entire data will need to be made freely accessible if your manuscript is accepted for publication. This policy applies to all data except where public deposition would breach compliance with the protocol approved by your research ethics board. If you are unable to adhere to our open data policy, please kindly revise your statement to explain your reasoning and we will seek the editor's input on an exemption. Please be assured that, once you have provided your new statement, the assessment of your exemption will not hold up the peer review process.

5. We note you have included a table to which you do not refer in the text of your manuscript. Please ensure that you refer to Table 1, 2, 3 in your text; if accepted, production will need this reference to link the reader to the Table.

6. Please include captions for your Supporting Information files at the end of your manuscript, and update any in-text citations to match accordingly. Please see our Supporting Information guidelines for more information: http://journals.plos.org/plosone/s/supporting-information.

7. Please remove all personal information, ensure that the data shared are in accordance with participant consent, and re-upload a fully anonymized data set.

Note: spreadsheet columns with personal information must be removed and not hidden as all hidden columns will appear in the published file.

Additional guidance on preparing raw data for publication can be found in our Data Policy (https://journals.plos.org/plosone/s/data-availability#loc-human-research-participant-data-and-other-sensitive-data) and in the following article: http://www.bmj.com/content/340/bmj.c181.long.

8. If the reviewer comments include a recommendation to cite specific previously published works, please review and evaluate these publications to determine whether they are relevant and should be cited. There is no requirement to cite these works unless the editor has indicated otherwise.

Additional Editor Comments :

Manuscript ID: PONE-D-25-67419

Title: Perceptions and Practices Among Students, Parents, and Teachers of the Human Papilloma Virus (HPV) Vaccine: A Pilot Feasibility Study from Rural Kerala

Corresponding Author: Anjum John, MD, MPH

Date of Review: January 10, 2026

Overall Assessment

This manuscript presents a pilot feasibility study exploring HPV vaccine-related knowledge, attitudes, and practices (KAP) among students, teachers, and parents in rural Kerala. The topic is of high public health relevance, particularly in light of India’s planned national HPV vaccine rollout. The study is framed appropriately as a pilot, with clear feasibility objectives. However, the manuscript suffers from significant methodological and ethical shortcomings, incomplete reporting, and overinterpretation of limited data. In its current form, it does not meet the standards for publication in PLOS ONE.

Decision: Major Revision Required

Detailed Review by Section

1. Title and Abstract

• Title: Clear but contains a typographical error (“Human Papilloma Virus (HPV)”). Correct spacing and punctuation are needed.

• Abstract:

o Appropriately summarizes the pilot’s purpose and main findings.

o However, it fails to mention the critical limitation of zero parent participation, which fundamentally affects the study’s feasibility conclusions.

o The statement “multivariable analysis was not undertaken” is misleading, it implies it was considered but omitted due to data, rather than being inherently outside the scope of a pilot.

o Recommendation: Explicitly state the absence of parent data and clarify that the pilot’s goal was tool/process refinement, not hypothesis testing.

2. Introduction

• Provides a comprehensive background on HPV, cervical cancer burden, and vaccination challenges in India and Kerala.

• Literature review is adequate, though somewhat dense. Could be streamlined to focus more on the KAP evidence gap in school settings.

• Rationale is clearly stated: to pilot a questionnaire and assess feasibility for a larger study.

3. Methods

3.1. Study Design and Setting

• Appropriately described as a pilot cross-sectional study.

• However, study dates are inconsistent: data collection is noted as June 2025 in the methods, but submission is dated December 2025. This suggests either prospective dating or a clerical error that needs correction.

3.2. Ethics

• Ethical approval is noted, but the consent process is problematic.

o Parental consent was sought but not obtained because “parents were unavailable.” This is a major flaw, parental participation was a key study aim. The researchers should have implemented a follow-up mechanism (e.g., phone calls, rescheduled meetings).

o Verbal assent from students is acceptable, but details of how assent was documented are missing.

3.3. Questionnaire Development

• Describes content validation, pilot testing, and Cronbach’s alpha, which is good.

• However, the questionnaire itself is not provided in the submission (neither in main text nor supplementary). This is essential for reproducibility.

• Cronbach’s alpha is reported as ≥0.7, but no actual value or breakdown by subscale (knowledge, attitude, practice) is given.

3.4. Data Collection

• Paper-based forms were used, but no information on data quality checks or inter-rater reliability is provided.

• The statement that “no sociodemographic information was collected” is a major methodological weakness for a KAP study, even as a pilot. Socioeconomic and educational factors are key predictors of vaccine awareness and uptake.

4. Results

4.1. Participant Flow

• Zero parent participation is a critical finding that should dominate the results and discussion of feasibility. It is currently underemphasized.

• Student and teacher demographics are provided, but the gender imbalance (78.7% female students, 90% female teachers) limits generalizability and may bias attitude responses.

4.2. KAP Findings

• Tables 2-4 are clear and useful.

• However, results are presented without measures of uncertainty (no confidence intervals, no tests of difference between students and teachers).

• The authors note “no inferential analysis,” but even descriptive statistics should include percentages with denominators clearly stated (some totals in tables do not sum correctly).

4.3. “Significant Misconceptions” Section

• This is interpretive and belongs in the Discussion, not Results.

• Phrases like “poor understanding,” “major gaps,” and “low-to-moderate” are subjective without benchmarking against validated thresholds.

5. Discussion

• Overinterprets pilot data as if from a full-scale analytical study.

• Compares findings to national and global literature appropriately, but fails to contextualize the pilot’s severe limitations (single school, no parents, no sociodemographics).

• The section on “Proposed Changes to Questionnaire” is useful and appropriate for a pilot, but it is buried in the Discussion. It should be moved to a dedicated “Lessons Learned for Future Research” section.

6. Public Health Implications and Strengths/Limitations

• Strengths: Correctly identifies feasibility focus and tool validation.

• Limitations: Understates the impact of missing parent data and lack of sociodemographic variables.

• The limitation regarding “self-reported practices” is noted, but not the social desirability bias inherent in vaccine attitude surveys in school settings.

7. Ethical and Reporting Compliance

• Ethics Statement: Provided, but the consent lapse with parents must be explicitly addressed as a limitation and a lesson learned.

• Data Availability: States data are “available upon request” due to privacy, which does not comply with PLOS ONE’s data policy. De-identified data must be deposited in a public repository or provided as supplementary files.

• Funding: Correctly declared as none.

8. Presentation and Language

• Writing: Generally clear but contains grammatical errors and informal phrasing (e.g., “vaccine shyness”).

• Tables: Well-structured but some formatting inconsistencies (e.g., missing totals, alignment).

• References: Extensive but some formatting issues (e.g., inconsistent journal abbreviations).

Major Revisions Required

1. Address Parent Non-Participation: This must be framed as a central feasibility finding, not just a deviation. Discuss why it happened and how it will be rectified in the main study.

2. Provide the Questionnaire: Include the full questionnaire as a supplementary file.

3. Deposit Data: Share de-identified data in a public repository or as supporting information.

4. Clarify Study Dates: Correct the temporal discrepancy between data collection (June 2025) and submission (December 2025).

5. Strengthen Methods: Describe how assent was documented and detail data quality control procedures.

6. Restructure Discussion: Separate “lessons learned for tool refinement” from interpretive comparison with other studies. Avoid overgeneralization.

Minor Revisions

1. Correct title punctuation: “Human Papillomavirus (HPV) Vaccine”.

2. Report exact Cronbach’s alpha value and subscale reliabilities.

3. Add confidence intervals or inter-group comparisons where appropriate.

4. Move “Significant Misconceptions” from Results to Discussion.

5. Proofread for language consistency and academic tone.

Final Comments

This pilot study addresses an important topic and appropriately focuses on feasibility and tool refinement. However, the manuscript in its current form lacks the methodological rigor, ethical completeness, and data transparency required for publication in PLOS ONE. With substantial revisions, particularly regarding parent participation, data sharing, and questionnaire transparency, the study could provide a valuable foundation for future research.

I recommend major revision and look forward to reviewing a revised version that addresses these critical concerns.

Signed,

Prof. Morufu Olalekan Raimi

[Note: HTML markup is below. Please do not edit.]

[NOTE: If reviewer comments were submitted as an attachment file, they will be attached to this email and accessible via the submission site. Please log into your account, locate the manuscript record, and check for the action link "View Attachments". If this link does not appear, there are no attachment files.]

To ensure your figures meet our technical requirements, please review our figure guidelines: https://journals.plos.org/plosone/s/figures

You may also use PLOS’s free figure tool, NAAS, to help you prepare publication quality figures: https://journals.plos.org/plosone/s/figures#loc-tools-for-figure-preparation.

NAAS will assess whether your figures meet our technical requirements by comparing each figure against our figure specifications.

# Author comment: Author response to Decision Letter 1

**DOI:** 10.1371/journal.pone.0340559.r002

5 Jun 2026

Date: June 5, 2026

From "Anjum John" dranjumsjohn@pimsrc.edu.in;johnanj@gmail.com

To : "PLOS ONE" plosone@plos.org

Subject: PLOS ONE Decision: Revision required [PONE-D-25-67419]

PONE-D-25-67419

Perceptions and Practices Among Students, Parents, and Teachers of the Human Papilloma Virus( HPV) vaccine : A Pilot Feasibility Study from Rural Kerala

Dear Editors and Reviewers,

Thank you for reviewing our manuscript in such detail and your efforts to make the manuscript as descriptive and informative as possible for readers. Thank you for the merit you see in this pilot study and encouragement to publish a pilot study with its methodological flaws reported in its completeness. We agree that our work was not perfect, and we have learned from this experience. Accordingly, we are submitting a revised version of the manuscript that addresses the points raised during the review process. Thank you for accommodating our request for extensions of the deadline for submission of the revised manuscript.

We are including the following:

1. A letter that responds to each point raised by the academic editor and reviewer(s). This letter is uploaded as a separate file labelled “ Response to Reviewers”.

2. A marked-up copy of your manuscript that highlights changes made to the original version. This is uploaded as a separate file labeled 'Revised Manuscript with Track Changes'.

3. An unmarked version of the revised paper without tracked changes. This is uploaded as a separate file labeled 'Manuscript'.

No changes have been made to our financial disclosure, and hence no statement has been updated in our cover letter. Please do note that we have no funding for this research or publication as our institution does not support any such funding.

Kind regards,

Anjum John, Corresponding author

Specific comments and replies

Journal Requirements:

When submitting your revision, we need you to address these additional requirements.

1. Please ensure that your manuscript meets PLOS ONE's style requirements, including those for file naming. The PLOS ONE style templates can be found at https://journals.plos.org/plosone/s/file?id=wjVg/PLOSOne_formatting_sample_main_body.pdf and https://journals.plos.org/plosone/s/file?id=ba62/PLOSOne_formatting_sample_title_authors_affiliations.pdf

We have done our best to ensure that our manuscript meets PLOS One’s style requirements.

2. Please include your full ethics statement in the ‘Methods’ section of your manuscript file. In your statement, please include the full name of the IRB or ethics committee who approved or waived your study, as well as whether or not you obtained informed written or verbal consent. If consent was waived for your study, please include this information in your statement as well.

Our full ethics statement has been included in the “Methods” section of the manuscript file. The full name of the IRB, which approved the study and details about the informed consent have been included.

3. In the online submission form, you indicated that the data cannot be shared publicly because of privacy reasons but they are available with the authors and will be provided anonymously, upon reasonable request. All PLOS journals now require all data underlying the findings described in their manuscript to be freely available to other researchers, either 1. In a public repository, 2. Within the manuscript itself, or 3. Uploaded as supplementary information.

This policy applies to all data except where public deposition would breach compliance with the protocol approved by your research ethics board. If your data cannot be made publicly available for ethical or legal reasons (e.g., public availability would compromise patient privacy), please explain your reasons on resubmission and your exemption request will be escalated for approval.

Response : Thank you for highlighting the journal's data availability requirements.

The dataset underlying this pilot study contains individual-level survey responses collected from school-going adolescents aged 11–15 years and teachers. Although direct identifiers were removed during data entry, the combination of demographic and response variables from a single-school pilot study conducted in a small rural setting may create a risk of indirect participant identification. Participant confidentiality therefore remains an important ethical consideration.

To comply with the journal's data-sharing policy while protecting participant privacy, we have prepared a fully anonymized dataset with all direct identifiers removed and with potentially identifying variables appropriately minimized. This anonymized dataset will be provided as Supporting Information accompanying the manuscript. No information that could reasonably be used to identify individual participants has been included.

We have revised the Data Availability Statement accordingly and trust that this approach satisfies the journal's requirements for transparency and reproducibility while maintaining participant confidentiality.

4. When completing the data availability statement of the submission form, you indicated that you will make your data available on acceptance. We strongly recommend all authors decide on a data sharing plan before acceptance, as the process can be lengthy and hold up publication timelines. Please note that, though access restrictions are acceptable now, your entire data will need to be made freely accessible if your manuscript is accepted for publication. This policy applies to all data except where public deposition would breach compliance with the protocol approved by your research ethics board. If you are unable to adhere to our open data policy, please kindly revise your statement to explain your reasoning and we will seek the editor's input on an exemption. Please be assured that, once you have provided your new statement, the assessment of your exemption will not hold up the peer review process.

Response to Comment 4:

Thank you for this clarification. We agree with the journal's recommendation to establish a data-sharing plan prior to acceptance. To comply with the journal's Open Data Policy, we have revised our Data Availability Statement. A fully anonymized dataset underlying the findings reported in this manuscript will be provided as Supporting Information and will be freely accessible upon publication. All direct identifiers have been removed, and the dataset has been carefully reviewed to minimize the risk of participant identification while preserving the integrity of the analyses.

We believe this approach balances transparency and reproducibility with our ethical obligation to protect participant confidentiality, particularly given that the study involved adolescent school students. The revised Data Availability Statement has been updated accordingly in the manuscript and submission system.

5. We note you have included a table to which you do not refer in the text of your manuscript. Please ensure that you refer to Table 1, 2, 3 in your text; if accepted, production will need this reference to link the reader to the Table.

Response to Comment 5:Thank you for bringing this to our attention. The table summarizing the key findings at the end of the manuscript was not explicitly cited in the text. To address this issue and avoid redundancy with the main results tables, we have moved this summary table to the Supporting Information as Supporting Information S4 Table.( Summary of key findings and implications from the pilot feasibility study on HPV vaccination knowledge, attitudes, and practices.) All remaining tables are now cited sequentially within the main text.

6. Please include captions for your Supporting Information files at the end of your manuscript, and update any in-text citations to match accordingly. Please see our Supporting Information guidelines for more information: http://journals.plos.org/plosone/s/supporting-information

Response : Captions for Supporting Information files have been included to the end of the manuscript and in-text citations have been updated to match, with reference to the Supporting Information Guidelines of the journal.

7. Please remove all personal information, ensure that the data shared are in accordance with participant consent, and re-upload a fully anonymized data set.

Response : The shared data set has all personal information removed and data is shared in accordance with participant consent, and a fully anonymized dataset has been re-uploaded. Spreadsheet columns with personal information have been removed and not hidden as directed.

Additional guidance on preparing raw data for publication can be found in our Data Policy (https://journals.plos.org/plosone/s/data-availability#loc-human-research-participant-data-and-other-sensitive-data) and in the following article: http://www.bmj.com/content/340/bmj.c181.long.

8. If the reviewer comments include a recommendation to cite specific previously published works, please review and evaluate these publications to determine whether they are relevant and should be cited. There is no requirement to cite these works unless the editor has indicated otherwise.

Response : Thank you.

Additional Editor Comments :

Manuscript ID: PONE-D-25-67419

Title: Perceptions and Practices Among Students, Parents, and Teachers of the Human Papilloma Virus (HPV) Vaccine: A Pilot Feasibility Study from Rural Kerala

Corresponding Author: Anjum John, MD, MPH

Date of Review: January 10, 2026

Overall Assessment

This manuscript presents a pilot feasibility study exploring HPV vaccine-related knowledge, attitudes, and practices (KAP) among students, teachers, and parents in rural Kerala. The topic is of high public health relevance, particularly in light of India’s planned national HPV vaccine rollout. The study is framed appropriately as a pilot, with clear feasibility objectives. However, the manuscript suffers from significant methodological and ethical shortcomings, incomplete reporting, and overinterpretation of limited data. In its current form, it does not meet the standards for publication in PLOS ONE.

Decision: Major Revision Required

Detailed Review by Section

1. Title and Abstract

• Title: Clear but contains a typographical error (“Human Papilloma Virus (HPV)”). Correct spacing and punctuation are needed.

Response : We thank the reviewer for these valuable observations. We have corrected the title to read “Human Papillomavirus (HPV)” throughout the manuscript.

• Abstract:

o Appropriately summarizes the pilot’s purpose and main findings.

o However, it fails to mention the critical limitation of zero parent participation, which fundamentally affects the study’s feasibility conclusions.

o The statement “multivariable analysis was not undertaken” is misleading, it implies it was considered but omitted due to data, rather than being inherently outside the scope of a pilot.

o Recommendation: Explicitly state the absence of parent data and clarify that the pilot’s goal was tool/process refinement, not hypothesis testing.

Response : We thank the reviewer for these valuable observations. We agree that the absence of parent participation is an important finding from a feasibility perspective and have now explicitly reported this limitation in the Abstract. The Abstract has been revised to explicitly report the absence of parent participation, clarify the feasibility-focused objectives of the pilot study, and remove the implication that multivariable analyses were planned but omitted. The Conclusions have also been revised to emphasize methodological insights gained from the pilot.

2. Introduction

• Provides a comprehensive background on HPV, cervical cancer burden, and vaccination challenges in India and Kerala.

• Literature review is adequate, though somewhat dense. Could be streamlined to focus more on the KAP evidence gap in school settings.

• Rationale is clearly stated: to pilot a questionnaire and assess feasibility for a larger study.

Response:

We thank the reviewer for the positive assessment of the Introduction and rationale. In response to the suggestion, we have streamlined portions of the literature review to reduce background detail and place greater emphasis on the existing evidence gaps regarding HPV-related knowledge, attitudes, and practices among school-based stakeholders, particularly students, parents, and teachers. The rationale for the pilot study has also been further clarified to emphasize its feasibility and questionnaire-development objectives.

3. Methods

3.1. Study Design and Setting

• Appropriately described as a pilot cross-sectional study.

• However, study dates are inconsistent: data collection is noted as June 2025 in the methods, but submission is dated December 2025. This suggests either prospective dating or a clerical error that needs correction.

Comment : We thank the reviewer for this observation. We have carefully reviewed the manuscript and confirmed that data collection was conducted between 18 and 19 June 2025, as stated in the Methods section. The manuscript was subsequently prepared and submitted after completion of data collection and analysis. We were unable to identify any conflicting study dates within the manuscript text and believe the reviewer may be referring to the manuscript submission date recorded in the journal system. To avoid any ambiguity, we have reviewed and clarified all study dates in the revised manuscript.

3.2. Ethics

• Ethical approval is noted, but the consent process is problematic.

o Parental consent was sought but not obtained because “parents were unavailable.” This is a major flaw; parental participation was a key study aim. The researchers should have implemented a follow-up mechanism (e.g., phone calls, rescheduled meetings).

o Verbal assent from students is acceptable, but details of how assent was documented are missing.

Comment : We thank the reviewer for this important observation. We acknowledge that the absence of parental participation represents a significant limitation of the study. However, as this investigation was designed as a pilot feasibility study, one of its objectives was to identify practical challenges associated with recruitment and study implementation. The inability to obtain parent participation despite distributing information sheets and questionnaires through students highlighted an important feasibility barrier that will inform the design of the larger study.

We agree that additional recruitment strategies such as follow-up telephone calls, parent meetings, reminder notices, or rescheduled data collection sessions could improve parental participation. These approaches have now been acknowledged in the Discussion and incorporated into the proposed modifications for the full-scale study.

Regarding student assent, verbal assent was obtained from all participating students after providing age-appropriate information about the study. Investigators maintained a study log documenting assent procedures and participant enrolment. Additional details regarding the assent process have now been added to the Methods section for clarity.

3.3. Questionnaire Development

• Describes content validation, pilot testing, and Cronbach’s alpha, which is good.

• However, the questionnaire itself is not provided in the submission (neither in main text nor supplementary). This is essential for reproducibility.

Response: We thank the reviewer for this valuable suggestion. We agree that providing the questionnaire enhances transparency, reproducibility, and the utility of the study for future researchers. Accordingly, the complete questionnaire used in this pilot study has now been included as Supporting Information (Supplementary File S1). Appropriate references to the supplementary questionnaire have been added in the Methods section.

• Cronbach’s alpha is reported as ≥0.7, but no actual value or breakdown by subscale (knowledge, attitude, practice) is given.

Response : Thank you for this observation. We agree that reporting only a threshold value was insufficient. We have revised the manuscript to provide the actual Cronbach's alpha coefficients obtained during preliminary pilot testing of the questionnaire. The overall questionnaire demonstrated acceptable internal consistency (Cronbach's α = 0.

Attachment Submitted filename: HPV Manuscript reply to Editors.docx

# Editor report: Decision Letter 1

**DOI:** 10.1371/journal.pone.0340559.r003

**Authors:** Morufu Raimi

11 Jun 2026

PONE-D-25-67419R1

Perceptions and Practices Among Students, Parents, and Teachers of the Human Papillomavirus (HPV) Vaccination: A Pilot Feasibility Study from Rural Kerala

PLOS One

Dear Dr. John,

Thank you for submitting your manuscript to PLOS ONE. After careful consideration, we feel that it has merit but does not fully meet PLOS ONE’s publication criteria as it currently stands. Therefore, we invite you to submit a revised version of the manuscript that addresses the points raised during the review process.

Please submit your revised manuscript by Jul 26 2026 11:59PM. If you will need more time than this to complete your revisions, please reply to this message or contact the journal office at plosone@plos.org. When you're ready to submit your revision, log on to https://www.editorialmanager.com/pone/ and select the 'Submissions Needing Revision' folder to locate your manuscript file.

Please include the following items when submitting your revised manuscript:

- A letter that responds to each point raised by the academic editor and reviewer(s). You should upload this letter as a separate file labeled 'Response to Reviewers'.
- A marked-up copy of your manuscript that highlights changes made to the original version. You should upload this as a separate file labeled 'Revised Manuscript with Track Changes'.
- An unmarked version of your revised paper without tracked changes. You should upload this as a separate file labeled 'Manuscript'.

If you would like to make changes to your financial disclosure, please include your updated statement in your cover letter. Guidelines for resubmitting your figure files are available below the reviewer comments at the end of this letter.

If applicable, we recommend that you deposit your laboratory protocols in protocols.io to enhance the reproducibility of your results. Protocols.io assigns your protocol its own identifier (DOI) so that it can be cited independently in the future. For instructions see: https://journals.plos.org/plosone/s/submission-guidelines#loc-laboratory-protocols. Additionally, PLOS ONE offers an option for publishing peer-reviewed Lab Protocol articles, which describe protocols hosted on protocols.io. Read more information on sharing protocols at https://plos.org/protocols?utm_medium=editorial-email&utm_source=authorletters&utm_campaign=protocols.

As the corresponding author, your ORCID iD is verified in the submission system and will appear in the published article. PLOS supports the use of ORCID, and we encourage all coauthors to register for an ORCID iD and use it as well. Please encourage your coauthors to verify their ORCID iD within the submission system before final acceptance, as unverified ORCID iDs will not appear in the published article. *Only*  the individual author can complete the verification step; PLOS staff *cannot*  verify ORCID iDs on behalf of authors.

We look forward to receiving your revised manuscript.

Kind regards,

Morufu Olalekan Raimi, Ph.D

Academic Editor

PLOS One

**Journal Requirements:**

1. If the reviewer comments include a recommendation to cite specific previously published works, please review and evaluate these publications to determine whether they are relevant and should be cited. There is no requirement to cite these works unless the editor has indicated otherwise.

2. Please review your reference list to ensure that it is complete and correct. If you have cited papers that have been retracted, please include the rationale for doing so in the manuscript text, or remove these references and replace them with relevant current references. Any changes to the reference list should be mentioned in the rebuttal letter that accompanies your revised manuscript. If you need to cite a retracted article, indicate the article’s retracted status in the References list and also include a citation and full reference for the retraction notice.

**Additional Editor Comments:**

PLOS ONE Editorial Decision

Manuscript ID: PONE-D-25-67419R1

Title: Perceptions and Practices Among Students, Parents, and Teachers of the Human Papillomavirus (HPV) Vaccination: A Pilot Feasibility Study from Rural Kerala

Corresponding Author: Dr. Anjum John

Editor: Prof. Morufu Olalekan Raimi, PhD

Date of Decision: June 11, 2026

Decision: Minor Revision

Executive Summary

The authors have conducted a thorough and responsive revision addressing the major methodological, ethical, and reporting concerns raised in the prior review. The manuscript is substantially improved: the abstract now correctly frames the pilot’s feasibility objectives and explicitly reports zero parent participation; the questionnaire and anonymized dataset are provided as supplementary files; Cronbach’s alpha values are reported with subscale breakdowns; interpretive material has been moved from Results to Discussion; and a dedicated “Lessons Learned for Future Research” section has been added. The authors have also moderated overgeneralizations and strengthened limitations regarding gender imbalance, single-school setting, and social desirability bias. However, despite these commendable improvements, several residual issues require attention before the manuscript can be accepted for publication. These are minor but necessary corrections to ensure clarity, consistency, and compliance with journal standards.

Minor Revisions

1. Inconsistent numerical data in Tables (Critical for reproducibility)

• Table 2 (Knowledge): For K4 (HPV vaccine most effective before sexual debut), student responses show “Yes 38 (50.7)” and “No 37” – but 38+37 = 75, yet the percentage for “Yes” is correctly 50.7%. Please verify and align all such pairs to avoid any perception of rounding errors.

• Table 2, K11: “Do you know where to get the HPV vaccine?” – Student “Yes” = 41 (54.7%?) but denominator is 75. Current text shows “Yes 41, No 34” (sum 75). Please recalculate percentage and ensure consistency with the 95% CI added in the revised manuscript (which currently shows different values in the track-changes version vs. clean version).

• Table 4, P2: “Have you ever received the HPV vaccine?” For students, “Yes 39 (52.0%), No 36 (48.0%)” – correct. But the table in the clean manuscript (page 54) erroneously repeats “39 (52.0%)” twice under the teacher column. Teacher responses should be “Yes 10 (50.0%), No 10 (50.0%)” as shown elsewhere. Please correct.

2. Missing in-text citation for Supplementary Table S4

• In the clean manuscript (page 37), you state that the summary table was moved to Supporting Information as S4 Table. However, there is no in-text citation to S4 Table anywhere in the revised manuscript. Please add a citation (e.g., in the Discussion or Conclusions) to direct readers to this summary.

3. Redundant text in the “Practices Related to HPV Vaccination” section

• Page 54 (clean manuscript), Table 4 is immediately followed by a second, incomplete Table 4 fragment. Please delete the duplicate fragment beginning with “Table 4. HPV vaccine awareness…” that appears after the correct table.

4. Typographical and grammatical corrections

• Page 22 (abstract): Remove the extra comma: “major gaps in HPV-related knowledge, attitudes, and practices, among stakeholders” → delete comma after “practices.”

• Page 31 (Discussion): “The study suggests that the understanding … remain incomplete” → “remains incomplete” (subject-verb agreement).

• Page 32: “Aslemash et al.” should be “Asmelash et al.” (consistent with reference 40).

• Page 35 (Lessons Learned, section a): The list begins with “1. Add socioeconomic…” but subsequent sub-sections use “b., c., d.” – change “1.” to “a.” for consistency.

• Page 60 (reference 18): Remove the extra space and period: “5133–8. .” → “5133–8.”

5. Data Availability Statement

• The statement says data are provided as Supporting Information. Please confirm that the anonymized dataset file has been uploaded with the revision and is cited as a supplementary file (e.g., S5 File or similar). Currently, I see references to S1-S4 Tables but no reference to a separate data file in the manuscript text.

6. Minor formatting inconsistency

• In the clean manuscript, some section headings (e.g., “Materials and methods” on page 25) use sentence case, while others (e.g., “Results” on page 27) use title case. Please standardize to PLOS ONE style (title case for main sections).

Recommendation

The manuscript now meets the essential standards for a pilot feasibility study report in PLOS ONE. The topic is timely and relevant, the revisions have been responsive and good-faith, and the lessons learned will genuinely inform future research. Once the above minor corrections are completed, the manuscript will be acceptable for publication.

I therefore recommend: Minor Revision.

No further statistical or methodological review is required. I look forward to receiving the final corrected version.

Signed,

Prof. Morufu Olalekan Raimi

Academic Editor, PLOS ONE

[Note: HTML markup is below. Please do not edit.]

[NOTE: If reviewer comments were submitted as an attachment file, they will be attached to this email and accessible via the submission site. Please log into your account, locate the manuscript record, and check for the action link "View Attachments". If this link does not appear, there are no attachment files.]

To ensure your figures meet our technical requirements, please review our figure guidelines: https://journals.plos.org/plosone/s/figures

You may also use PLOS’s free figure tool, NAAS, to help you prepare publication quality figures: https://journals.plos.org/plosone/s/figures#loc-tools-for-figure-preparation.

NAAS will assess whether your figures meet our technical requirements by comparing each figure against our figure specifications.

# Author comment: Author response to Decision Letter 2

**DOI:** 10.1371/journal.pone.0340559.r004

12 Jun 2026

Dear Editors,

Thank you for the detailed review of our manuscript and the time spent on looking through the manuscript multiple times to ensure it is an article that is as error-free as possible. Thank you for handholding us through the entire process. We have tried to make all the changes suggested to our manuscript and hope this meets the requirements of the Editors and the Journal.

We have included the following items with the revised manuscript:

• A letter that responds to each point raised by the academic editor and reviewer(s) has been included and labelled 'Response to Reviewers'.

• A marked-up copy of your manuscript that highlights changes made to the original version is uploaded as a separate file labelled 'Revised Manuscript with Track Changes'.

• An unmarked version of your revised paper without tracked changes is uploaded this as a separate file labelled 'Manuscript'.

1. Decision: Minor Revision

Executive Summary

The authors have conducted a thorough and responsive revision addressing the major methodological, ethical, and reporting concerns raised in the prior review. The manuscript is substantially improved: the abstract now correctly frames the pilot’s feasibility objectives and explicitly reports zero parent participation; the questionnaire and anonymized dataset are provided as supplementary files; Cronbach’s alpha values are reported with subscale breakdowns; interpretive material has been moved from Results to Discussion; and a dedicated “Lessons Learned for Future Research” section has been added. The authors have also moderated overgeneralizations and strengthened limitations regarding gender imbalance, single-school setting, and social desirability bias. However, despite these commendable improvements, several residual issues require attention before the manuscript can be accepted for publication. These are minor but necessary corrections to ensure clarity, consistency, and compliance with journal standards.

Response: We thank the reviewer for this helpful observation and for their positive assessment of the revisions made to the manuscript. We agree that this issue required clarification. Accordingly, we have revised the manuscript and relevant changes have been incorporated in the revised manuscript.

Minor Revisions

1. Inconsistent numerical data in Tables (Critical for reproducibility)

• Table 2 (Knowledge):

a. For K4 (HPV vaccine most effective before sexual debut), student responses show “Yes 38 (50.7)” and “No 37” – but 38+37 = 75, yet the percentage for “Yes” is correctly 50.7%. Please verify and align all such pairs to avoid any perception of rounding errors.

b. Table 2, K11: “Do you know where to get the HPV vaccine?” – Student “Yes” = 41 (54.7%?) but denominator is 75. Current text shows “Yes 41, No 34” (sum 75). Please recalculate percentage and ensure consistency with the 95% CI added in the revised manuscript (which currently shows different values in the track-changes version vs. clean version).

Response: We thank the reviewer for this careful review of the numerical data and for highlighting these inconsistencies.

a. K4 (HPV vaccine most effective before sexual debut)

We rechecked the original dataset and verified that the correct student responses for K4 are: Yes = 35 (46.7%) and No = 40 (53.3%), which sum to the total sample of 75 students. The values referred to by the reviewer (Yes = 38 and No = 37) were present in an earlier tracked-changes version and do not reflect the final dataset. We have carefully reviewed the table and ensured that the frequencies, percentages, and corresponding 95% confidence intervals are consistent throughout the revised manuscript and supplementary materials.

b. K11 (Do you know where to get the HPV vaccine?)

We thank the reviewer for identifying this issue. Upon verification against the original dataset, the correct student responses are: Yes = 40 (53.3%) [41.4–64.9] and No = 35 (46.7%) [35.1–58.6], based on a denominator of 75 students. The discrepancy noted by the reviewer arose from differences between an earlier tracked-changes version and the final clean version of the manuscript. We have rechecked all frequencies, percentages, and 95% confidence intervals in Table 2 and confirmed that the values reported in the revised manuscript are correct and internally consistent.

In addition, we conducted a comprehensive review of all tables, percentages, confidence intervals, and supplementary files to ensure complete consistency and reproducibility across the manuscript.

•Table 4, P2: “Have you ever received the HPV vaccine?” For students, “Yes 39 (52.0%), No 36 (48.0%)” – correct. But the table in the clean manuscript (page 54) erroneously repeats “39 (52.0%)” twice under the teacher column. Teacher responses should be “Yes 10 (50.0%), No 10 (50.0%)” as shown elsewhere. Please correct.

Response:

We thank the reviewer for identifying this error and are very sorry this was not picked up during our review of the manuscript.

Upon verification against the original dataset, we confirmed that a typographical error occurred in Table 4 (P2: “Have you ever received the HPV vaccine?”). The teacher column incorrectly repeated the student value “39 (52.0%) [40.2–63.6]”. The correct teacher responses are:

Yes = 10 (50.0%) [27.2–72.8]

No = 10 (50.0%) [27.2–72.8]

The table has been corrected accordingly in the revised manuscript. We have also rechecked all frequencies, percentages, and confidence intervals reported in Table 4 against the original dataset to ensure accuracy and internal consistency.

2. Missing in-text citation for Supplementary Table S4

• In the clean manuscript (page 37), you state that the summary table was moved to Supporting Information as S4 Table. However, there is no in-text citation to S4 Table anywhere in the revised manuscript. Please add a citation (e.g., in the Discussion or Conclusions) to direct readers to this summary.

Response: Thank you- we have added a statement towards the end of the first paragraph of the Discussion to reflect this. “A summary of the study findings is presented in Supporting Information Table S4.”

3. Redundant text in the “Practices Related to HPV Vaccination” section

• Page 54 (clean manuscript), Table 4 is immediately followed by a second, incomplete Table 4 fragment. Please delete the duplicate fragment beginning with “Table 4. HPV vaccine awareness…” that appears after the correct table.

Response: We are very sorry this happened and we have removed the half-table that followed the Table 4 in the clean document.

4. Typographical and grammatical corrections

• Page 22 (abstract): Remove the extra comma: “major gaps in HPV-related knowledge, attitudes, and practices, among stakeholders” → delete comma after “practices.”

Comments: The corrected sentence has been highlighted in the manuscript. Thank you.

• Page 31 (Discussion): “The study suggests that the understanding … remain incomplete” → “remains incomplete” (subject-verb agreement).

Response: Thank you, the correction has been made.

• Page 32: “Aslemash et al.” should be “Asmelash et al.” (consistent with reference 40).

Response: Thank you, the correction has been made.

• Page 35 (Lessons Learned, section a): The list begins with “1. Add socioeconomic…” but subsequent sub-sections use “b., c., d.” – change “1.” to “a.” for consistency.

Response: Thank you, the correction has been made.

• Page 60 (reference 18): Remove the extra space and period: “5133–8.” → “5133–8.”

Response: Thank you, the correction has been made.

5. Data Availability Statement

• The statement says data are provided as Supporting Information. Please confirm that the anonymized dataset file has been uploaded with the revision and is cited as a supplementary file (e.g., S5 File or similar). Currently, I see references to S1-S4 Tables but no reference to a separate data file in the manuscript text.

Response: The anonymized data file is provided as an Excel sheet and though labelled as Supplementary file is attached in Excel Format as a separate file. (S3 File – Fully anonymized dataset)

6. Minor formatting inconsistency

• In the clean manuscript, some section headings (e.g., “Materials and methods” on page 25) use sentence case, while others (e.g., “Results” on page 27) use title case. Please standardize to PLOS ONE style (title case for main sections).

Response: Thank you, the correction has been made.

Recommendation

The manuscript now meets the essential standards for a pilot feasibility study report in PLOS ONE. The topic is timely and relevant, the revisions have been responsive and good-faith, and the lessons learned will genuinely inform future research. Once the above minor corrections are completed, the manuscript will be acceptable for publication.

Respected sir, thank you for your very gracious and thorough examination of our manuscript and the time taken to re-review it before final approval. We appreciate the time and effort you have put in to help make this manuscript even better.

Warm Regards,

Anjum

Date: June 12, 2026

Attachment Submitted filename: RE Review June 12 comments.docx

# Editor report: Decision Letter 2

**DOI:** 10.1371/journal.pone.0340559.r005

**Authors:** Morufu Raimi

21 Jun 2026

Perceptions and Practices Among Students, Parents, and Teachers of the Human Papillomavirus (HPV) Vaccination: A Pilot Feasibility Study from Rural Kerala

PONE-D-25-67419R2

Dear Authors,

We’re pleased to inform you that your manuscript has been judged scientifically suitable for publication and will be formally accepted for publication once it meets all outstanding technical requirements.

Within one week, you’ll receive an e-mail detailing the required amendments. When these have been addressed, you’ll receive a formal acceptance letter and your manuscript will be scheduled for publication.

An invoice will be generated when your article is formally accepted. Please note, if your institution has a publishing partnership with PLOS and your article meets the relevant criteria, all or part of your publication costs will be covered. Please make sure your user information is up-to-date by logging into Editorial Manager at Editorial Manager® and clicking the ‘Update My Information' link at the top of the page. For questions related to billing, please contact billing support.

If your institution or institutions have a press office, please notify them about your upcoming paper to help maximize its impact. If they’ll be preparing press materials, please inform our press team as soon as possible -- no later than 48 hours after receiving the formal acceptance. Your manuscript will remain under strict press embargo until 2 pm Eastern Time on the date of publication. For more information, please contact onepress@plos.org.

Kind regards,

Morufu Olalekan Raimi, Ph.D

Academic Editor

PLOS One

Additional Editor Comments (optional):

PLOS ONE Editorial Decision

Manuscript Number: PONE-D-25-67419R2

Title: Perceptions and Practices Among Students, Parents, and Teachers of the Human Papillomavirus (HPV) Vaccination: A Pilot Feasibility Study from Rural Kerala

Authors: John et al.

Editor: Dr. Morufu Olalekan Raimi

DECISION: Accept

DETAILED ASSESSMENT

I have completed the evaluation of the revised manuscript (R2). The authors have addressed all concerns raised in the previous review round thoroughly and professionally.

RESOLUTION OF PREVIOUS CONCERNS

1. Numerical Data Consistency: The authors have corrected all identified discrepancies in Tables 2 and 4. Verification against the original dataset confirms that frequencies, percentages, and 95% confidence intervals are now internally consistent and accurately reported. The correction of K4 to Yes=35 (46.7%) and No=40 (53.3%), and K11 to Yes=40 (53.3%) and No=35 (46.7%) resolves the summation errors. The teacher column in Table 4, P2 has been corrected to Yes=10 (50.0%) and No=10 (50.0%).

2. Supplementary Material Citations: The in-text citation for Supporting Information Table S4 has been added in the Discussion section, providing readers with appropriate direction to the summary of findings.

3. Redundant Text: The duplicate Table 4 fragment has been removed from the manuscript.

4. Typographical and Grammatical Corrections: All identified errors have been addressed, including the comma removal in the abstract, subject-verb agreement correction, author name spelling (“Asmelash et al.”), list formatting consistency, and reference formatting.

5. Data Availability Statement: The anonymized dataset has been confirmed as uploaded and cited as Supporting Information (S3 File), ensuring compliance with PLOS ONE data availability requirements.

6. Formatting Consistency: Section headings have been standardized to title case throughout the manuscript, aligning with journal style.

MANUSCRIPT STRENGTHS

This pilot feasibility study makes a valuable contribution to understanding HPV vaccine perceptions in a rural Indian context, a setting of significant public health importance given India's high cervical cancer burden and ongoing national HPV vaccine rollout. The study's primary strength lies in its transparent reporting of methodological challenges, particularly the complete absence of parental participation, which provides critical insights for future research design. The dedicated "Lessons Learned for Future Research" section is comprehensive and actionable, demonstrating the value of conducting this pilot before proceeding to a larger analytical study. The authors have appropriately framed the study as exploratory and feasibility-driven, consistently acknowledging limitations regarding generalizability, gender imbalance, single-school setting, and self-reported practices. The revisions have substantially improved manuscript clarity and methodological transparency.

MANUSCRIPT WEAKNESSES (appropriately acknowledged)

The absence of parental data limits the comprehensiveness of findings, and the predominance of female participants may influence the generalizability of results. The single-school setting and reliance on self-reported practices are recognized limitations that the authors have transparently addressed.

FINAL RECOMMENDATION

The authors have satisfactorily resolved all minor concerns from the previous review. The manuscript is methodologically transparent, appropriately cautious in its claims, and provides meaningful guidance for future HPV vaccination research in school settings. It meets PLOS ONE's publication standards.

I recommend acceptance of this manuscript in its current form.

Sincerely,

Dr. Morufu Olalekan Raimi

Academic Editor, PLOS ONE

# Editor report: Acceptance letter

**DOI:** 10.1371/journal.pone.0340559.r006

**Authors:** Morufu Raimi

PONE-D-25-67419R2

PLOS One

Dear Dr. John,

I'm pleased to inform you that your manuscript has been deemed suitable for publication in PLOS One. Congratulations! Your manuscript is now being handed over to our production team.

At this stage, our production department will prepare your paper for publication. This includes ensuring the following:

* All references, tables, and figures are properly cited

* All relevant supporting information is included in the manuscript submission,

* There are no issues that prevent the paper from being properly typeset

You will receive further instructions from the production team, including instructions on how to review your proof when it is ready. Please keep in mind that we are working through a large volume of accepted articles, so please give us a few days to review your paper and let you know the next and final steps.

Lastly, if your institution or institutions have a press office, please let them know about your upcoming paper now to help maximize its impact. If they'll be preparing press materials, please inform our press team within the next 48 hours. Your manuscript will remain under strict press embargo until 2 pm Eastern Time on the date of publication. For more information, please contact onepress@plos.org.

You will receive an invoice from PLOS for your publication fee after your manuscript has reached the completed accept phase. If you receive an email requesting payment before acceptance or for any other service, this may be a phishing scheme. Learn how to identify phishing emails and protect your accounts at https://explore.plos.org/phishing.

If we can help with anything else, please email us at customercare@plos.org.

Thank you for submitting your work to PLOS ONE and supporting open access.

Kind regards,

PLOS ONE Editorial Office Staff

on behalf of

Prof Morufu Olalekan Raimi

Academic Editor

PLOS One
